# Phylogenetic Analysis of the MS4A and TMEM176 Gene Families

**DOI:** 10.1371/journal.pone.0009369

**Published:** 2010-02-23

**Authors:** Jonathan Zuccolo, Jeremy Bau, Sarah J. Childs, Greg G. Goss, Christoph W. Sensen, Julie P. Deans

**Affiliations:** 1 Department of Biochemistry and Molecular Biology, University of Calgary, Calgary, Alberta, Canada; 2 Immunology Research Group, Institute of Infection, Immunity and Inflammation, Faculty of Medicine, University of Calgary, Calgary, Alberta, Canada; 3 Sun Center of Excellence for Visual Genomics, Faculty of Medicine, University of Calgary, Calgary, Alberta, Canada; 4 Department of Biological Sciences, University of Alberta, Edmonton, Alberta, Canada; 5 Bamfield Marine Sciences Centre, Bamfield, British Columbia, Canada; National Institute on Aging, United States of America

## Abstract

**Background:**

The MS4A gene family in humans includes CD20 (MS4A1), FcRβ (MS4A2), Htm4 (MS4A3), and at least 13 other syntenic genes encoding membrane proteins, most having characteristic tetraspanning topology. Expression of MS4A genes is variable in tissues throughout the body; however, several are limited to cells in the hematopoietic system where they have known roles in immune cell functions. Genes in the small TMEM176 group share significant sequence similarity with MS4A genes and there is evidence of immune function of at least one of the encoded proteins. In this study, we examined the evolutionary history of the MS4A/TMEM176 families as well as tissue expression of the phylogenetically earliest members, in order to investigate their possible origins in immune cells.

**Principal Findings:**

Orthologs of human MS4A genes were found only in mammals; however, MS4A gene homologs were found in most jawed vertebrates. TMEM176 genes were found only in mammals and bony fish. Several unusual MS4A genes having 2 or more tandem MS4A sequences were identified in the chicken (*Gallus gallus*) and early mammals (opossum, *Monodelphis domestica* and platypus, *Ornithorhyncus anatinus*). A large number of highly conserved MS4A and TMEM176 genes was found in zebrafish (*Danio rerio*). The most primitive organism identified to have MS4A genes was spiny dogfish (*Squalus acanthus*). Tissue expression of MS4A genes in *S. acanthias* and *D. rerio* showed no evidence of expression restricted to the hematopoietic system.

**Conclusions/Significance:**

Our findings suggest that MS4A genes first appeared in cartilaginous fish with expression outside of the immune system, and have since diversified in many species into their modern forms with expression and function in both immune and nonimmune cells.

## Introduction

The MS4A gene family in humans includes at least 16 genes that encode membrane proteins typically having tetraspanning topology [1], [2]. This family is relatively uncharacterized at the protein level, with some important exceptions: MS4A1 (CD20) is expressed exclusively in B lymphocytes, where the protein has a function in regulating calcium influx downstream of the activated B cell antigen receptor [3]–[6]. CD20 is the target of immunotherapeutic antibodies used to deplete pathogenic B cells in chronic lymphocytic leukemia, lymphomas, autoimmune diseases and in solid organ transplantation [7]–[10]. MS4A2 (FcRβ) is a signaling subunit of the high affinity IgE receptor (FcεRI) and the low affinity IgG receptor (FcγRIII) on mast cells, and therefore has a key role in hypersensitivity and allergic reactions [11]. MS4A3 (Htm4) is expressed on intracellular membranes of lymphoid and myeloid cells, functioning as an adaptor protein in cell cycle regulation [12]. MS4A4A has not been characterized in humans, but the mouse ortholog, MS4A4B, has restricted expression in T cells, where it appears to have a role in Th1 development, CD8+ memory T cell function and modulation of regulatory T cell signaling [13]–[16]. MS4A12 is restricted to colonic epithelial cells and, like CD20, functions in store operated calcium influx [17]. The functions of the remaining MS4A proteins are unknown, but it is possible that they comprise a family of ion channel/adaptor proteins [5], [11], [12], [17], [18]. Whereas expression of the first 4 members of the MS4A family is restricted to the hematopoietic system, MS4A5 and MS4A12 are restricted to testis and colon, respectively, and others are broadly expressed in various hematopoietic and nonhematopoietic tissues [1], [2], [17], [19].

All MS4A genes are clustered on chromosome 11q in humans [20] (chromosome 19 in mice) in a region with linkage to allergy and atopy [21]. Two MS4A related genes, TMEM176A and TMEM176B, are located on chromosome 7 (chromosome 6 in mice) [22]. The human TMEM176 proteins share up to 16% amino acid sequence identity with MS4A proteins and are of similar length and topology. Indeed, it has been suggested that TMEM176 genes are members of the MS4A family [22]. TMEM176B (LR8, Torid, Clast1) is broadly expressed but was up regulated in antigen presenting cells in a rat model of allograft tolerance [22]. However, the predominant phenotype of TMEM176B deficient mice is ataxia caused by impaired development of cerebellar granule cells [23]. TMEM176A is expressed in hepatic malignancies but has not been further characterized [24].

MS4A and TMEM176 genes have been identified and mapped in humans and mice, but have not been comprehensively analyzed in other species. The evolutionary history of these genes has not been examined and their first appearance during phylogeny is unknown. In this study, we identified all genes in currently available databases from species selected to cover large evolutionary distances. MS4A genes were found among most vertebrate groups, including mammals, birds, reptiles, amphibians, and jawed fish. TMEM176 genes are fewer than MS4A genes and were found in mammals and bony fish. The most primitive species with clear examples of MS4A genes was S*qualus acanthias* (spiny dogfish). We found no evidence of MS4A or TMEM176 genes in jawless vertebrates, protists, bacteria, fungi or plants. A group of potentially related genes was identified in several invertebrate species, however, regions of similarity were limited to very short sequences that upon further examination were also found in numerous unrelated membrane proteins.

The adaptive immune system, as defined by the central importance of immunoglobulin domain containing antigen receptors, has been identified only in jawed vertebrates and first appeared in cartilaginous fish such as sharks and rays [25], [26]. As mentioned above, several MS4A and TMEM176 proteins in mammals have roles in adaptive immune functions. This, together with identification of a cartilaginous fish as the earliest species with MS4A genes, suggested the possibility of their functional coevolution with adaptive immunity. However, transcriptional analysis of MS4A genes in tissues from S. *acanthias* indicated that the emergence of both adaptive immunity and MS4A genes in cartilaginous jawed fish was coincidental.

## Materials and Methods

### Sequence Information Retrieval

Reference Sequence and nucleotide databases were searched by tBLASTn using protein sequences corresponding to each of the human MS4A genes; this retrieved a large number of mammalian sequences as well as sequences from avian (*Gallus gallus* and *Taeniopygia guttata*) and bony fish (*Danio rerio* and *Osmerus mordax*) species. Similar searches conducted using the retrieved sequences from *G. gallus*, *T. guttata*, *D. rerio* and *O. mordax* yielded no additional sequences. Next, we searched the expressed sequence tag (EST) database using the protein sequence from *O. mordax*, retrieving sequences from the Eastern Tiger Salamander (*Ambystoma tigrinum tigrinum*), green anole lizard (*Anolis carolinensis*), Western clawed frog (*Xenopus tropicalis*), African clawed frog (*Xenopus laevis*), numerous species of bony fish, the cartilaginous spiny dogfish (*Squalus acanthias*), and several invertebrate species. The sequence from *S. acanthias* was then used to search the Reference Sequence, nucleotide and EST databases again, yielding no additional sequences, however a search of the RefSeq-RNA database retrieved sequences from the invertebrate lancelet (*Branchiostoma floridae*).

The Conserved Domain Database (CDD) [27], [28](http://www.ncbi.nlm.nih.gov/Structure/cdd/wrpsb.cgi) was searched using human CD20 as the original query protein sequence. CDD uses Reverse Position Specific BLAST (RPS BLAST) to find distant relatives of a protein based on conserved features in the sequences. The initial search identified 729 protein sequences from diverse species. Conserved Domain Architecture Retrieval Tool (CDART) [29] was then used to screen the 729 sequences. CDART scrutinizes the sequential order of the domains identified in order to increase the specificity of each match. CDART retrieved 419 protein sequences from the original dataset of 729 sequences. Pairwise alignment was used to compare a selection of eliminated sequences with known MS4A protein sequences to verify that CDART accurately removed nonconserved proteins. The remaining 419 sequences included vertebrate MS4A gene sequences overlapping with those found in the earlier searches, as well as sequences from several invertebrate species, including *B. floridae*.

Using selected sequences obtained from the searches described above, the genomes of the following species were specifically searched: human (*Homo sapiens*), chimp (*Pan troglodytes*), mouse (*Mus musculus*), rat (*Rattus norvegicus*), horse (*Equus caballus*), cow (*Bos taurus*), dog (*Canis lupus familiaris*), grey short-tailed opossum (*Monodelphis domestica*), platypus (*Ornithorhyncus anatinus*), chicken (*G. gallus*), zebrafinch (*T. guttata*), Western clawed frog (*X. tropicalis*), African clawed frog *(X. laevis)*, zebrafish *(D. rerio*), spotted green pufferfish (*Tetraodon nigroviridis*), Japanese pufferfish *(Takifugu rubripes*), elephant shark *(Callorhinchus milii*), purple sea urchin *(Strongylcentrotus purpuratus*), and a number of plant, invertebrate, and prokaryote genomes. Finally, short regions of conserved sequences identified by CDD were used to search EST databases of lampreys, hagfishes, lancelets, tunicates, cephalochordates and protostomes.

Multiple sequence alignment of all retrieved sequences was performed using ClustalX [30] to identify and remove duplicate, splice variant, reading frame shift, truncated or otherwise non usable MS4A sequences from the dataset. The authenticity of uncertain sequences was evaluated by searching the genomic and EST databases by tBLASTn using the identified sequences as the query and also by using transmembrane prediction software [31].

### Phylogenetic Analysis

An alignment of predicted MS4A and TMEM176 protein sequences from all species was produced using ClustalX. Genedoc [32] was used for manual editing of sequence alignments and shading of conserved residues. We used PHYLIP version 3.6a3 for our phylogenetic analysis [33]. SEQBOOT was used to create 1000 delete-half jackknife datasets and their subsequent bootstrap values from the sequence alignment. The distance analysis was performed using PROTDIST and subsequently NEIGHBOR with standard parameters. CONSENSE was used to determine the best phylogenetic tree. The trees were generated for presentation using FigTree v. 1.1.1 (http://tree.bio.ed.ac.uk/software/figtree/).

### Animal Care

Wildtype zebrafish embryos were raised and maintained according to Westerfield [34] with approval from the University of Calgary Animal Care Committee. The embryos were raised in 0.3 x E3 Solution. At 24 hours post fertilization, 0.003% phenylthiourea was added to the media to block pigment formation. The embryos were euthanized at 96 hours post fertilization using tricaine (3-amino benzoic acidethylester). Embryos were fixed using 4% paraformaldehyde in PBS. Pacific spiny dogfish (*S. acanthias* L.) were caught by hook and bait from the Trevor Channel (Vancouver Island, BC, Canada) and immediately transferred to the Bamfield Marine Sciences Centre, where they were held in batches in a 151,000 L circular tank provided with flowing seawater (13°C, 3.2% salinity). Dogfish were terminally anesthetized and tissues harvested according to University of Alberta approved animal care protocols.

### RNA Preparation and PCR Conditions

Spiny dogfish organs were obtained from wild caught specimens and flash frozen in liquid nitrogen prior to storage. Tissues were later weighed and ground up in a RNAse free mortar and pestle on dry ice. The resulting tissue powder was extracted with Trizol (Invitrogen, Carlsbad, CA) reagent according to the manufacturer instructions. 5 µg of total RNA was reverse transcribed using Superscript III (Invitrogen) with oligo-dT primers. Polymerase chain reaction (PCR) was performed in 25 µl volumes with TAQ PCR master mix (Qiagen, Valencia, CA) using 32 cycles of 1 min at 95°C, 1 min at 58°C, and 50 sec at 72°C. PCR primers used for Squ.A.MS4Ax1 forward - 5′ CAA TTC TCC TCA CCG TCG TT 3′ and Squ.A.MS4Ax1 reverse–5′ TGG CGT TCT TCA CTC TTC CT 3′. Primers used for Squ.A.MS4Ax2 forward–5′ GGC TAC AAT CGT CCC TCA AA 3′ and reverse–5′ AGC GGG TAT GCA GAA AAT TG 3′. Primers used for Squ.A.MS4Ax3 forward–5′ CCT CGG TAG CGA TTC AGA 3′ and reverse–5′ GGA GCG AAG GAA ATC ACA GA–3′. Primers for Squ.A.CD79A forward–5′ ATC GGG AGT GCG TTT CT GCT 3′ and reverse–5′ GGC TGT CGC TCA CCC TGT AG 3′. Primers for Squ.A.Beta Actin forward–5′ GAC TTT GAA CAG GAG ATG GC 3′ and reverse 5′ GCT CAG GTG GGG CAA TAA T -3′. PCR products were resolved on 1% agarose Tris-Borate-EDTA (TBE) gels with ethidium bromide and photographed to observe relative signals of each amplified transcript.

### Probe Synthesis and Whole-Mount *In Situ* Hybridization in Zebrafish

Antisense RNA probes for MS4A17A.17 were produced by polymerase chair reaction (PCR) using Taq Polymerase followed by RNA synthesis using T7 Polymerase (Promega, Madison, WI) and the following primer sequences: forward–5′ GGT CAA CAA TCC TTT AAT TCA ACA C 3′ and reverse–5′ AAT TTA ATA CGA CTC ACT ATA GGA TAT ACA CAT AGG TAA TGG CAA AAC TT 3′ (T7 Promoter region underlined). Digoxygenin (DIG) labeled RNA probes were synthesized and *in situ* hybridization was then carried out according to Thisse et al. (1993) [35] with several changes: the glycine rinse was omitted and the embryos were incubated at 65°C. The staining was stopped by a wash with 4% PFA in PBS and two washes in PBT. Wholemount embryos were mounted in in 3% methylcellulose and visualized using Zeiss Stemi SV11 equipped with an Axiocam HRc. For sectioning, embryos were then dehydrated in a gradient of ethanol/water and infiltrated and catalyzed with JB4 medium (Polysciences, Warrington, PA). 5 µm sections were cut using glass knives on a Leica microtome and imaging was performed with on a Leica DMR microscope equipped with an Optronics Magnafire camera.

## Results

### Identification of MS4A and TMEM176 Genes

**Figure 1:**
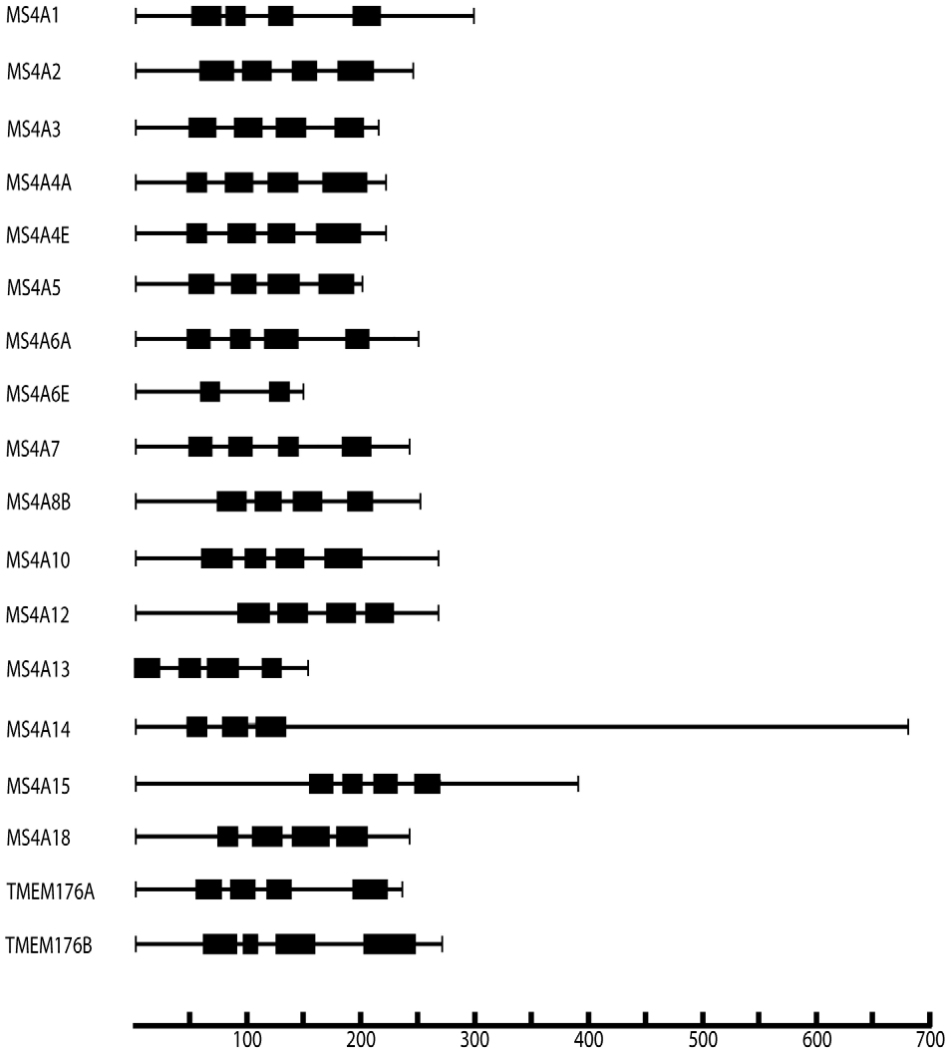
Predicted topology and length of human MS4A and TMEM176 proteins. Line diagrams are drawn to scale. Thick lines represent transmembrane domains. Thin lines represent intracellular and extracellular regions. N termini are predicted to localize in the cytoplasm.

Previous reports have described 12 human MS4A genes: MS4A1, MS4A2, MS4A3, MS4A4A, MS4A4E, MS4A5, MS4A6A, MS4A6E, MS4A7, MS4A8B, MS4A10 and MS4A12, as well as 2 MS4A related genes, TMEM176A and TMEM176B. In this study, we found three unreported human MS4A genes in the Genbank database–MS4A13, MS4A14 and MS4A15, as well as one gene that had not previously been identified as a MS4A family member that we named MS4A18. Analysis of the human genome revealed that the latter gene is located in the same region of chromosome 11 as the other MS4A genes. Line diagrams of the human MS4A protein sequences are shown in **Figure 1**. Most MS4A proteins include 200 to 300 amino acids with 4 transmembrane spans; MS4A6E, MS4A13, MS4A14 and MS4A15 are exceptions. MS4A6E appears to be a truncated MS4A gene with only 2 transmembrane domains [2]. MS4A13 is unusual in having a truncated N terminal cytoplasmic domain. MS4A14 is over twice as long as other MS4A proteins, with an extended sequence at the C terminus. MS4A15 has somewhat longer cytoplasmic regions than most other MS4A proteins but is otherwise typical.

Using the search strategies described in the methods, MS4A genes were found in human (*H. sapiens*), chimpanzee (*P. troglodytes*), macaque (*M. mulatta*), orangutan (*P. abelii*), horse (*E. caballus*), cow (*B. taurus*), dog (*C. familiaris*), cat (F. *catus*), pig (*S. scrofa*), sheep (*O. aries*), mouse (*M. musculus*), rat (*R. norvegicus*), marmoset (*C. jacchus*), gerbil (*M. unguiculatus*), grey short-tailed opossum (*M. domestica*), duck-billed platypus (*O. anatinus*), chicken (*G. gallus*), zebrafinch (*T. guttata*), Western clawed frog (*X. tropicalis*), African clawed frog (*X. laevis*), Eastern Tiger Salamander (*A. tigrinum*), green anole lizard (*A. carolinensis*), numerous bony fish including zebrafish (*D. rerio*), spotted green pufferfish (*T. nigroviridis*), rainbow smelt (*O. mordax*) and Atlantic salmon (*S. salar*), and the spiny dogfish (*S. acanthias*) TMEM176 genes were found in mammalian and some bony fish species. Genes in representative species are listed in **Table S1**, organized to indicate orthology.

Mammalian species with sequenced genomes, including the platypus *(O. anatinus)* and grey short-tailed opossum (*M. domestica*), were all found to encode several conserved MS4A genes (**Table S1**). Several distinct genes were also identified in the genome of spotted green pufferfish (*T. nigroviridis*) (**Table S1**). However, not all fish have MS4A genes. Neither elephant shark (*C. milii*), a cartilaginous fish related to, but evolutionarily distinct from sharks, or fugu, the Japanese pufferfish (*T. rubripes*), have MS4A or TMEM176 genes encoded in their genomes. Remarkably, 21 MS4A genes and 4 TMEM176 genes were identified in the zebrafish (*D. rerio*) genome (**Table S1**). In contrast, only 1 MS4A gene was found in each of the sequenced bird genomes (*G. gallus* and *T. guttata)*, and 2 in the genome of the frog, *X. tropicalis* (**Table S1**). No clearly identifiable MS4A or TMEM176 genes were found in the EST database and/or genomes of jawless vertebrates, invertebrates, plants, or bacteria. Combined, these results suggest that the MS4A gene family first appeared in the earliest jawed vertebrates from which the gene family expanded and diversified significantly.

### Phylogeny of the MS4A and TMEM176 Gene Families

Phylogenetic analysis was performed using MS4A and TMEM176 sequences from selected vertebrate species spanning large evolutionary distances. Mammalian species included were human, cow, mouse, opossum and platypus. The single MS4A sequence from the zebrafinch (*T. guttata*) was included to represent aves; this gene is nearly identical to part of the chicken MS4A gene, which is otherwise unusual as described below. Also included were all MS4A sequences found in reptilian and amphibian species (3 from *A. carolinensis*, 2 from *X. tropicalis* and 1 from *A. tigrinum*), all sequences from zebrafish (*D. rerio*) and spotted green pufferfish (*T. nigroviridis*), a representative sequence from other bony fish (*O. mordax*), and the single full length sequence identified in the spiny dogfish (*S. acanthias*). A list of all sequences used for phylogenetic analysis is provided in **Table S2**. Excluded from the phylogeny for the sake of clarity were genes from closely related mammalian species and a small group of exceptionally long sequences that were analyzed separately (see below).

**Figure 2:**
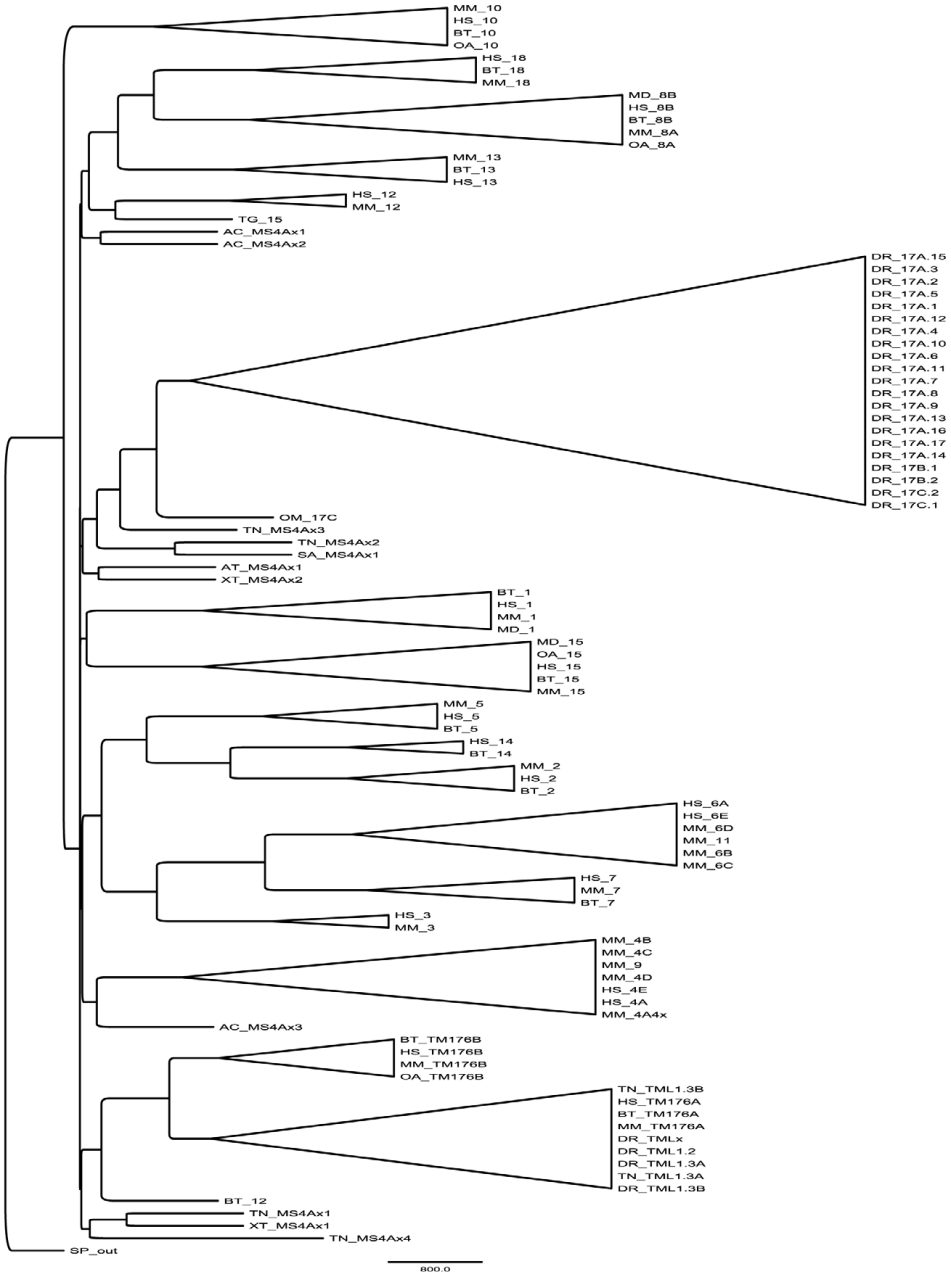
Phylogram of MS4A and TMEM176 families. Using the multiple sequence alignment shown in Supplementary Figure S1, a phylogeny was generated of all sequences listed in Supplementary Table S2, using a sequence from *S. purpuratus* to root the tree (SP_out). The expanded phylogenetic tree with bootstrap values is shown in supplementary Figure S2.

The phylogenetic tree was generated from sequences aligned as shown in **Figure S1**, using a sequence from purple sea urchin (*S. purpuratus*) as an outgroup (**Fig. S2**). A simplified version of this tree, with highly similar sequences merged, is shown in **Figure 2**. Notably, the 21 zebrafish (*D. rerio*) MS4A genes grouped into a single highly related cluster, suggesting that they diversified relatively recently from a common ancestral gene. In contrast, all mammalian species have several distinct MS4A genes with a high degree of sequence variability (**Table S1** and **Fig. 2**). MS4A genes found in most other bony fish are more similar to the zebrafish MS4A genes than to mammalian genes (**Fig. 2** and data not shown); the spiny dogfish MS4A gene appears at an ancestral point within this grouping of fish MS4A genes suggesting a common ancestral gene for all fish (**Fig. 2** and **Fig. S2**). Amphibians and reptiles are vertebrate groups intermediate between fish and mammals; MS4A genes from representative species (*X. tropicalis, A. tigrinum*, *A. carolinensis*) appear at multiple positions in the phylogenetic tree where they are found as an ancestral node to various sub groupings (**Fig. 2** and **Fig. S2**).

The TMEM176 genes, which were found on a separate chromosome from the MS4A genes in all genomes examined, appear on the phylogram as a group of genes less related to MS4A than to each other (**Fig. 2**) and display only partial sequence similarity with MS4A proteins (**Fig. S1**), suggesting early divergence from a common ancestor.

Several genes possibly related to MS4A/TMEM176 were identified in invertebrate species, particularly the lancelet (*B. floridiae*), sea anemone (*Nematostella vectensis*), sea squirt (*Ciona intestinalis*) and sea urchin (*S. purpuratus*). Alignment of these sequences with vertebrate MS4A genes showed some sequence similarity in the transmembrane domains. These short regions of similarity were also found in transmembrane domains of many unrelated multi-spanning proteins in diverse species, including examples from plants, eukaryotes, fungi, and archaea (not shown). Based on this finding, and also the lack of MS4A like sequences from jawless vertebrates, we conclude that the invertebrates lack MS4A or MS4A-like genes.

### Long MS4A Genes with Tandem MS4A Sequences

**Figure 3:**
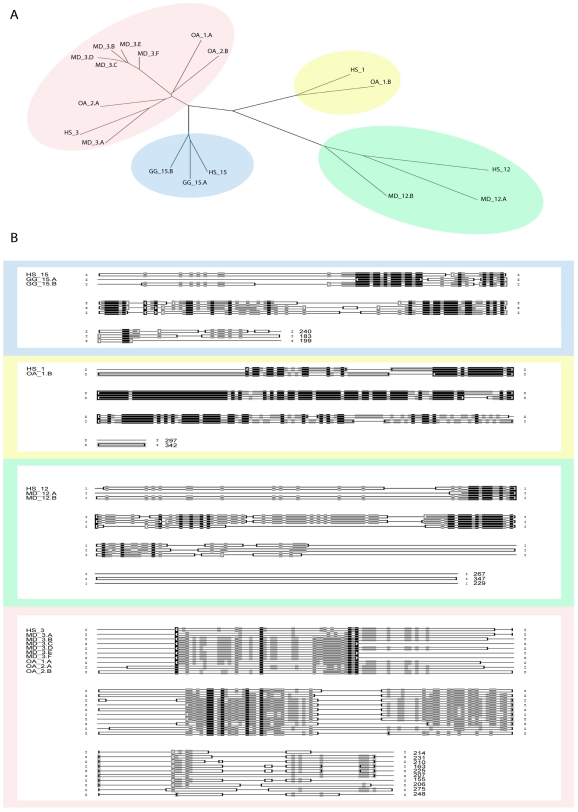
Tandem MS4A genes in *G. gallus*, *M. domestica* and *O. anatinus*. The long MS4A protein sequences were cut into their subunit components and renamed with the suffix A through F to denote their respective position relative to the amino terminus. Alignment and phylogeny of these sequences was performed using ClustalX. The detailed alignments are shown in supplementary Figure S3. A. An unrooted phylogeny of the MS4A subunit sequences compared to full length human MS4A sequences. B. A schematic representation of the sequence alignments was generated using Genedoc. Shading indicates the degree of sequence conservation. Black shading indicates that the amino acid is conserved; grey indicates partial conservation and white indicates there is little to no sequence conservation at the indicated position.

Most MS4A genes encode proteins up to 300 amino acids in length. In the entire dataset, 7 genes were identified that were significantly longer. Of these, human and bovine MS4A14 appear to be otherwise typical MS4A genes with exceptionally long C terminal regions. The remaining 5 genes were found in chicken (*G. gallus)*, opossum *(M. domestica)* and platypus (*O. anatinus)*. PSI BLAST searches using the corresponding protein sequences identified the presence of 2 or more tandem MS4A sequences in each polypeptide. With the exception of the MD_3 gene, which was identified in a single genomic clone, all of the tandem MS4A genes were identified in multiple unique genomic sequence clones suggesting these are not the product of artificial fusion events. To further examine the composition of the long MS4A proteins, each sequence was cut into its component subunits that were then compared to one another and to individual human MS4A sequences by pairwise alignment. A multiple sequence alignment of the subunits and the most closely related human MS4A protein sequences was used to generate an unrooted phylogenetic tree (**Fig. 3a**). Groupings of similar MS4A subunits, identified as major branches on the phylogram, were aligned in their individual groups (**Fig. 3b** **and Fig. S3**). The MS4A gene in the chicken (*G.gallus*) was found to encode 2 subunits that were both most similar to human MS4A15. One of the 2 long MS4A genes in the platypus (*O. anatinus*) contained 2 subunits that were both most similar to MS4A3. The second long platypus gene also contained 2 subunits, one of which was most similar to MS4A1 whereas the other was most similar to MS4A3. One of the 2 long MS4A genes in the opossum (*M. domestica*) contained 2 subunits that were both most similar to human MS4A12. The second long opossum gene was particularly remarkable, having 6 subunits that were all most similar to human MS4A3.

### MS4A and TMEM176 Gene Families in Zebrafish (*D. rerio*)

The large number of MS4A and TMEM176 genes in *D. rerio* warranted further analysis. Whereas mammalian MS4A genes are diverse in sequence yet clustered on a single chromosome, the 21 MS4A genes were found to be dispersed over 3 chromosomes, with the majority clustered on chromosome 4 (not shown). The remaining MS4A genes were found on chromosomes 5 and 15. With the exception of MS4A17A.17 (originally named MS4A4), the zebrafish genes found during the analysis were unnamed. The MS4A genes were therefore named as a new constituent gene group, MS4A17, because they are all more related to one another than to existing MS4A gene groups (**Fig. 2** and **Fig. S2**). The TMEM176 genes in zebrafish appear to be less related to TMEM176 genes found in mammals (**Fig. S2**) and were therefore named TMEM176 like genes (TMEM176l). Four of the 6 TMEM176l genes were found to be clustered on chromosome 16, with the remaining genes existing alone on chromosomes 7 and 1.

**Figure 4:**
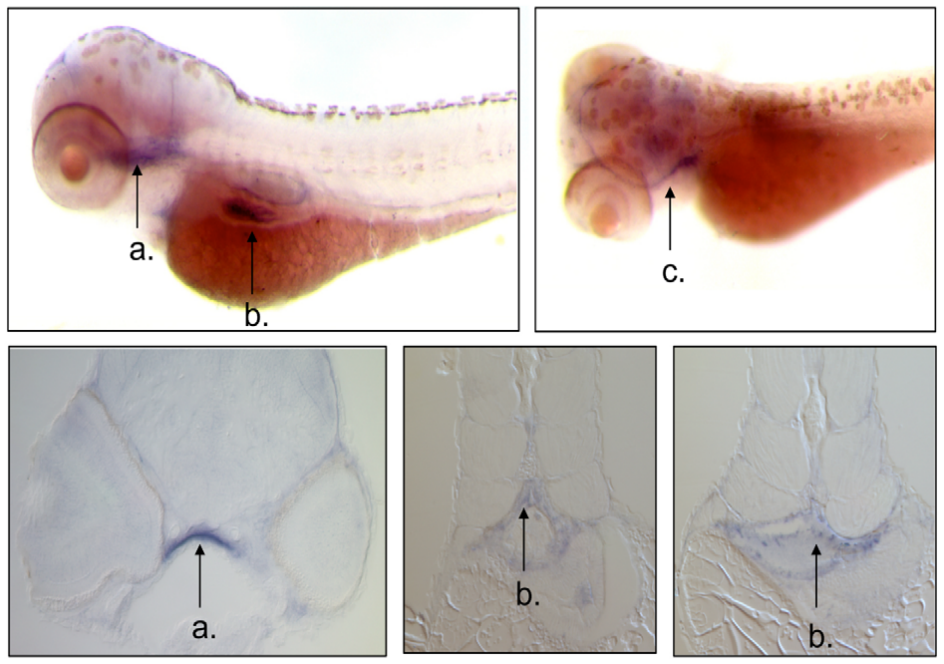
Tissue expression of MS4A17A.17 in *D. rerio*. Whole mount embryos (upper panels) and coronal sections (lower panels) of *in situ* hybridization of MS4A17A.17 probe in zebrafish 96 hours post fertilization embryo. Probe hybridized to the dorsal pharynx (arrow a in upper and lower left panels), an unidentified neuron (arrow c in upper right panel), and the mesenchyme surrounding the kidney glomerulus (arrow b in the upper left, lower middle and lower right panels).

Most of the MS4A genes in zebrafish are represented in EST databases constructed from whole zebrafish, indicating their expression at least at the level of mRNA. To directly examine tissue expression of a selected gene, we performed whole mount *in situ* hybridization in the 96 hours post fertilization zebrafish using a probe designed to detect MS4A17A.17 RNA. MS4A17A.17 appears to be expressed in the dorsal pharynx of the zebrafish head, the mesenchyme surrounding the kidney glomerulus, and in an unidentified neuron (**Fig. 4**); there was no evidence of expression in sites of hematopoiesis.

### MS4A Gene Expression in Spiny Dogfish *(S. acanthias)*

**Figure 5:**
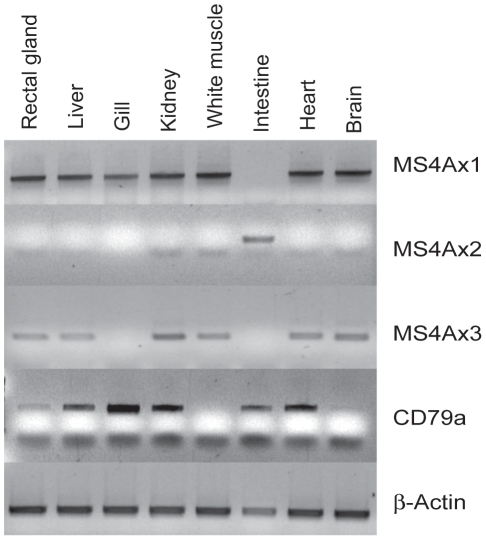
Tissue expression of MS4A genes in *S. acanthus*. Reverse transcriptase PCR was performed on RNA isolated from the tissues indicated, using primers to amplify each of the 3 distinct MS4A genes identified in the *S. acanthus* EST database. CD79a is a B lymphocyte expressed gene used to detect the presence of blood cells in each tissue or organ examined. Beta actin was used as a standardizing gene.

The earliest species in which MS4A genes were identified is *S. acanthias*, a representative of the first class of vertebrates having adaptive immune systems based on rearranged immunoglobulin domain containing genes that give rise to antigen binding receptors on T and B lymphocytes. In order to test the hypothesis that MS4A genes coevolved with the adaptive immune system, we examined MS4A mRNA expression by reverse transcriptase polymerase chain reaction in tissues and organs of spiny dogfish. We reasoned that, if the first appearance of MS4A genes and adaptive immunity in cartilaginous fish was purposeful, we should find at least one gene with expression restricted to blood cells. In addition to the full length sequence included in the phylogenetic analysis, fragments of 2 other distinct MS4A genes were identified. Each of the 3 spiny dogfish MS4A genes was found to have a unique expression profile among the tissues examined (**Fig. 5**). MS4Ax1 was found in all tissues except the intestine, whereas MS4Ax2 expression was found only in the intestine. MS4Ax3 was found in all tissues except the intestine and the gill. To determine the extent to which the tissues examined were infiltrated by blood cells, we analyzed expression of CD79a mRNA (a B lymphocyte marker) in each of the tissues. We found that CD79a was expressed in most tissue samples, but was completely absent in white muscle and only trace amounts were detected in the brain. Since MS4Ax1 and MS4Ax3 were detected in muscle and brain tissues, but CD79a was not, their expression is clearly not restricted to blood cells of the spiny dogfish. Furthermore, since CD79a was detected in intestinal tissue but MS4Ax1 and MS4Ax3 were not, these MS4A genes are unlikely to be expressed in the blood. The lack of expression of MS4Ax2 in several tissues with strong CD79a expression indicates that this MS4A gene is not expressed in blood cells. Based on these data, the known MS4A genes in the spiny dogfish are highly unlikely to be involved in adaptive immune functions.

## Discussion

We have analyzed available sequence data in order to better understand the origin of the MS4A and TMEM176 genes, several of which encode proteins with known functions in adaptive immunity. MS4A genes were found in all classes of jawed vertebrates but not in jawless vertebrates or phylogenetically earlier species, suggesting the possibility of their functional coevolution with adaptive immunity. However, tissue expression of MS4A genes in *S. acanthias*, the earliest vertebrate with MS4A genes, showed no evidence of expression in the hematopoietic system, leading to our conclusion that the appearance of MS4A genes and adaptive immunity in early vertebrate phylogeny was coincidental.

Several distinct MS4A genes were found in all mammalian genomes. Orthologs of human MS4A genes were found only in other mammals (**Table S1**). Representatives of marsupials and monotremes, the earliest mammals, were found to have fewer MS4A genes than placental mammals, but these MS4A genes were clear orthologs of human genes. In contrast, MS4A genes in lower vertebrates are generally fewer, less diverse, and highly divergent from mammalian MS4A genes. A large number of MS4A genes were found in the zebrafish, but these were highly related to one another and grouped separately from MS4A genes in other vertebrate classes, as did the MS4A genes identified in other bony and cartilaginous fish. In addition to the sequences shown in **Figure S1**, EST databases of other Cypriniformes fish (carp, goldfish, and loaches) also contain evidence of MS4A genes highly conserved with those of zebrafish (not shown). Sequencing of the spiny dogfish genome is incomplete, but evidence of 3 distinct MS4A genes was obtained from the EST database. The lack of MS4A genes in elephant shark, a member of a sister-group related to modern sharks, could be due to insufficient genomic sequence coverage, gene loss, extreme divergence of the gene sequences, or because the MS4A genes first appeared in modern sharks. We found no evidence of MS4A genes in jawless fish. A group of potentially related genes was identified in several invertebrate species; however, regions of similarity were limited to very short sequences that upon further examination were also found in numerous unrelated membrane proteins in diverse species including plants and microorganisms. The apparent lack of MS4A or MS4A like genes in the earliest vertebrates suggests that the invertebrate genes may be examples of convergent evolution rather than evidence of a common ancestral gene that gave rise to vertebrate MS4A genes and the identified invertebrate genes. Combined, the data suggest that chondrichthyes are the class where MS4A genes first appeared in their modern form, however, limited genomic and cDNA derived sequence data for agnathans (jawless fish) leaves open the possibility that MS4A genes may exist in these early vertebrates.

In the course of this investigation we identified a new MS4A gene in the human genome (MS4A18) and a new MS4A4 gene in the mouse (MS4A4x). Blast searches against EST databases revealed that expression of MS4A18 is limited to testis and that MS4A4x is expressed in osteoclast like cells (not shown). Previously, TMEM176 genes were identified and proposed to be related to MS4A genes [22]. We confirmed this relationship by phylogenetic analysis and sequence comparison. TMEM176 sequences have regions of highly conserved sequence with MS4A. However, several points suggest that TMEM176 genes are a separate group: 1) TMEM176 genes were found on a different chromosome than MS4A genes in all species examined; 2) sequence conservation between MS4A genes and TMEM176 genes in all species is relatively low; 3) phylogenetic analysis suggests that all TMEM176 genes are more related to each other than to any MS4A gene examined. Therefore, the TMEM176 genes presumably share a common ancestral gene with the MS4A gene family but diverged and underwent independent expansion.

Our identification of a few exceptionally long MS4A genes with tandem sequences was unexpected. These genes were found in the chicken and primitive mammals, but not in placental mammals or in fish or amphibian species. Homo-oligomerization of human CD20 (MS4A1) and hetero-oligomerization of mouse MS4A4B and MS4A6B have been reported [6], [13]. Discovery of tandem MS4A genes in chicken, opossum and platypus supports the concept that oligomerization may be a general feature of MS4A proteins. With the exception of MD_3, which contains 6 repetitive subunits, all of the long MS4A proteins we identified have a predicted structure of 2 tandem MS4A proteins suggesting that MS4A proteins may function as dimers or multiples of dimers. Indeed, CD20 assembles into tetramers that are probably organized as dimer of dimers [3], [6]. In the chicken and early mammals it appears that gene duplication/fusion events may have diversified the MS4A genes and their functional protein products while conserving genomic DNA.

In this study, we initially set out to determine the evolutionary origin of the MS4A and TMEM176 families, which include several genes encoding proteins with restricted expression in the immune system. In light of evidence that MS4A genes first appeared in cartilaginous fish (chondrichthyes), the earliest vertebrates with a modern form of adaptive immunity, we tested the hypothesis that MS4A genes evolved with the immune system by examining tissue expression of the 3 identified MS4A genes in the spiny dogfish. The results demonstrated that expression of all 3 genes is not restricted to blood cells. Furthermore, based on data that showed lack of expression of MS4A genes in tissues with strong evidence of blood cell infiltration, it appears that these genes may not be expressed in blood cells at all. However, it is possible that other, unidentified MS4A genes in the spiny dogfish are expressed in blood cells, or that any of these genes may be expressed in non-circulating lymphocytes in peripheral lymphoid tissues. With these caveats in mind, our findings suggest that MS4A genes first appeared in cartilaginous fish with expression in nonimmune cells, and have expanded and diversified in mammalian species into their modern forms with diverse functions in both immune and nonimmune cells.

## Supporting Information

Table S1 MS4A and TMEM176 homolog and ortholog distribution throughout phylogeny.
(0.03 MB XLS)

Table S2 MS4A and TMEM176 genes in species used for phylogenetic analysis.
(0.03 MB XLS)

Figure S1 Multiple sequence alignment of MS4A and TMEM176 proteins corresponding to genes listed in Table S2.
(1.17 MB PDF)

Figure S2 Phylogenetic tree of vertebrate MS4A and TMEM176 sequences. The tree was generated from the multiple alignment shown in Figure S1 using a sequence from *S. purpuratus* to root the tree (SP_out). Numbers represent bootstrap values out of 1000.
(1.26 MB EPS)

Figure S3 Alignment of subunits of long MS4A sequences. The long MS4A protein sequences were cut into their subunit components designated with the suffix A through F to denote their position relative to the amino terminus. Alignment of these subunits and human MS4A protein sequences was performed using ClustalX.
(0.12 MB PDF)
