# Supplementary material for: Phylogenetic Analysis of the MS4A and TMEM176 Gene Families
Source: PLoS One. 2010 Feb 23;5(2):e9369. doi: 10.1371/journal.pone.0009369 (PMC2826416; doi:10.1371/journal.pone.0009369)
Supplement: Figure S1 — Multiple sequence alignment of MS4A and TMEM176 proteins corresponding to genes listed in Table S2. (1.17 MB PDF) [file pone.0009369.s003.pdf]

```
                         *        20         *        40         *        60         *        80         *       100         *       12
MM_10      : ------------------------------------------------------------------------------------------------------------------MAGQA :   5
HS_10      : ------------------------------------------------------------------------------------------------------------------MKAEA :   5
BT_10      : ----------------------------------------------------------------------------------------------------------MAAAANEASTVTA :  13
OA_10      : ----------------------------------------------------------------------------------------------------------------MANEDNC :   7
HS_18      : --------------------------------------------------------MAFKT--LFSCPSIPLP-TMSKSQLSSLSVNGSYLELTKFRHPFTHSSNAKHLPLQQLYTMTE :  60
BT_18      : --------------------------------------------------------MMEQGVGANTVPGVTAPGNVYMIQARNPVAPGSKGQPVG-MATYLTSR-VTESDAGRANLQTP :  61
MM_18      : --------------------------------------------------------MSNQ----ENVSAVTVPSNVQVMQPTYAVVSGSHEQPLGKTTTYPASATMLQYNPGSANFQNP :  59
MD_8B      : -------------------------------------------------------------------------------------------------MTSNPEINNVFQVPPSTDSSMV :  22
HS_8B      : ------------------------------------------------------------------------------------------------------------------MNSMT :   5
BT_8B      : ------------------------------------------------------------------------------------------------------------------MNPTT :   5
MM_8A      : --------------------------------------------------------------------------------------------------MEPEQERLTRQPGTVSMNTVT :  21
OA_8A      : ---------------------------------------------------------------------------------------------------------------------MT :   2
MM_13      : ----------------------------------------------------------------------------------------------------------------------- :   -
BT_13      : ----------------------------------------------------------------------------------------------------------------------- :   -
HS_13      : ----------------------------------------------------------------------------------------------------------------------- :   -
HS_12      : --------------------------------------------------------------------------------MMSSKPTSHAEVNETIPNPY----PPGSFMAPGFQQPLG :  35
MM_12      : -----------------------------------------------------------------------------------------MTSSQTTYPGTRG-IPNPC----PPTRSMA :  25
TG_MS4A15  : ----------------------------------------------------------------------------------------------------------------------- :   -
AC_MS4Ax1  : -------------------------------------------------------------------------------------------------------------MASGTVVFLA :  10
AC_MS4Ax2  : ---------------------------------------------------------------------------------------------------------------------MA :   2
DR_17A.15  : ---------------------------------------------------------------------------------------------------------------------MS :   2
DR_17A.3   : ---------------------------------------------------------------------------------------------------------------------MS :   2
DR_17A.2   : ---------------------------------------------------------------------------------------------------------------------MA :   2
DR_17A.5   : ----------------------------------------------------------------------------------------------------------------------- :   -
DR_17A.1   : ----------------------------------------------------------------------------------------------------------------------- :   -
DR_17A.12  : ---------------------------------------------------------------------------------------------------------------------MS :   2
DR_17A.4   : ----------------------------------------------------------------------------------------------------------------------- :   -
DR_17A.10  : ---------------------------------------------------------------------------------------------------------------------MS :   2
DR_17A.6   : ----------------------------------------------------------------------------------------------------------------------- :   -
DR_17A.11  : ---------------------------------------------------------------------------------------------------------------------MS :   2
DR_17A.7   : ----------------------------------------------------------------------------------------------------------------------- :   -
DR_17A.8   : ---------------------------------------------------------------------------------------------------------------------MS :   2
DR_17A.9   : ----------------------------------------------------------------------------------------------------------------------- :   -
DR_17A.13  : ----------------------------------------------------------------------------------------------------------------------- :   -
DR_17A.16  : ---------------------------------------------------------------------------------------------------------------------ML :   2
DR_17A.17  : ---------------------------------------------------------------------------------------------------------------------MS :   2
DR_17A.14  : ---------------------------------------------------------------------------------------------------------------------MS :   2
DR_17B.1   : --------------------------------------------------------------------------------------------------------------------MSS :   3
DR_17B.2   : --------------------------------------------------------------------------------------------------------------------MSS :   3
DR_17C.2   : ----------------------------------------------------------------MTGVLVFLFGIVLTTSDYNYPNSSFSVISGLTYWGSFVYISAGSLSVAAQNKLHP :  55
DR_17C.1   : ----------------------------------------------------------------------------MKQNTIEFIISSF--ISGCAWKASYNRFHK-------HKQTPE :  34
OM_17C     : --------------------------------------------------------------------------------------------------------------------MSS :   3
TN_MS4Ax3  : ----------------------------------------------------------------------------------------------------------------------- :   -
TN_MS4Ax2  : ----------------------------------------------------------------------------------------------------------------------- :   -
SA_MS4Ax1  : ----------------------------------------------------------------------------------------------------------------------- :   -
AT_MS4Ax1  : ----------------------------------------------------------------------------------------------------------------------- :   -
XT_MS4Ax2  : ----------------------------------------------------------------------------------------------------------------------- :   -
BT_1       : ----------------------------------------------------------------------------------------------------------------------- :   -
HS_1       : ----------------------------------------------------------------------------------------------------------------------- :   -
MM_1       : ----------------------------------------------------------------------------------------------------------------MSGP--- :   4
MD_1       : ----------------------------------------------------------------------------------------------------------------------- :   -
MD_15      : ---------------------------------------------------------------------------------------------------------------------MT :   2
OA_15      : ---------------------------------------------------------------------------------------------------------------------MT :   2
HS_15      : ---------------------------------------------------------------------------------------------------------------------MS :   2
BT_15      : ---------------------------------------------------------------------------------------------------------------------MS :   2
MM_15      : ----------------------------------------------------------------------------------------MWERRGRGESAAGTAAVASRLRPPGSLNTMS :  31
MM_5       : ----------------------------------------------------------------------------------------------------------------------- :   -
HS_5       : ----------------------------------------------------------------------------------------------------------------------- :   -
BT_5       : ----------------------------------------------------------------------------------------------------------------------- :   -
HS_14      : ----------------------------------------------------------------------------------------------------------------------- :   -
BT_14      : ----------------------------------------------------------------------------------------------------------------------- :   -
MM_2       : ----------------------------------------------------------------------------------------------------------------------- :   -
HS_2       : ----------------------------------------------------------------------------------------------------------------------- :   -
BT_2       : ----------------------------------------------------------------------------------------------------------------------- :   -
HS_6A      : ----------------------------------------------------------------------------------------------------------------------- :   -
HS_6E      : ----------------------------------------------------------------------------------------------------------------------- :   -
MM_6D      : ----------------------------------------------------------------------------------------------------------------------- :   -
MM_11      : ----------------------------------------------------------------------------------------------------------------------- :   -
MM_6B      : ----------------------------------------------------------------------------------------------------------------------- :   -
MM_6C      : ----------------------------------------------------------------------------------------------------------------------- :   -
HS_7       : ----------------------------------------------------------------------------------------------------------------------- :   -
MM_7       : ----------------------------------------------------------------------------------------------------------------------- :   -
BT_7       : ----------------------------------------------------------------------------------------------------------------------- :   -
HS_3       : ----------------------------------------------------------------------------------------------------------------------- :   -
MM_3       : ----------------------------------------------------------------------------------------------------------------------- :   -
MM_4B      : ----------------------------------------------------------------------------------------------------------------------- :   -
MM_4C      : ----------------------------------------------------------------------------------------------------------------------- :   -
MM_9       : ----------------------------------------------------------------------------------------------------------------------- :   -
MM_4D      : ----------------------------------------------------------------------------------------------------------------------- :   -
HS_4E      : -----------------------------------------------------------------------------------------------------------------MGAVGE :   6
HS_4A      : ----------------------------------------------------------------------------------------------------------------------- :   -
MM_4x      : ----------------------------------------------------------------------------------------------------------------------- :   -
AC_MS4Ax3  : ---------------------------------------------------------------------------------------------------------------MSSGPMES :   8
BT_TM176B  : ---------------------------------------------------------------------------------------------------------------------MA :   2
HS_TM176B  : ---------------------------------------------------------------------------------------------------------------------MT :   2
MM_TM176B  : ---------------------------------------------------------------------------------------------------------------------MV :   2
OA_TM176B  : --------------------------------------------------------------------------------------------------------------MTDQVSGIP :   9
TN_TML1.3B : ----------------------------------------------------------------------------------------------------------------------- :   -
HS_TM176A  : ----------------------------------------------------------------------------------------------------------------------- :   -
BT_TM176A  : ----------------------------------------------------------------------------------------------------------------------- :   -
MM_TM176A  : ---------------------------------------------------------------------------------------------------------------------MS :   2
DR_TMLx    : ---------------------------------------------------------------------MNAIKQKQSHVIFTVDRLRRAIQTCHKSKETGDSAVVTCGKFITPVYNMP :  50
DR_TML1.2  : ---------------------------------------------------------------------------------------------------------------------MS :   2
DR_TML1.3A : MSVTVSHGEGTMIITVSSNPKSKWPMLCQILGSLCYGPVCFVPEDMRETLSSTQRVFGIVQIIFAILNLMLGCFGLIFSFKSCDLFYSSNTFGGQCNDVRVLFILQLIVAISFCAMTAK : 119
TN_TML1.3A : ---------------------------------------------------------------------------------------------------------------------MS :   2
DR_TML1.3B : ---------------------------------------------------------------------------------------------------------------------MS :   2
BT_12      : ----------------------------------------------------------------------------------------------------------------------- :   -
TN_MS4Ax1  : ----------------------------------------------------------------------------------------------------------------------- :   -
XT_MS4Ax1  : ----------------------------------------------------------------------------------------------------------------------- :   -
TN_MS4Ax4  : ----------------------------------------------------------------------------------------------------------------------- :   -
SP_out     : -----------------------------------------------------------MAEPPVYTPPEQQKTAYPPPGVYPQGQPAAYPQGQPGAYPQGQPVAYPQGQPVAYPQGQP :  60
```

```
               0         *       140         *       160         *       180         *       200         *       220         *       2
MM_10        : PTAVPGSVTG---EVSRWQNLGPA--------------------------------QPAQKVAQPQNLVPDGHL-------------------------------EKALEGSDLLQKLG :  58
HS_10        : TVIPSRCAR----GLPSWQVLSPV--------------------------------QPWQTSAPQNTTQPKLLAPHQHD--------------------------KSQKKSS-LLKELG :  61
BT_10        : RSEAEGHPSEKALSPTPPGLTDLP--------------------------------QKVTQPDLPPPNWHQ----------------------------------EKPRKSSRVLKELG :  66
OA_10        : RDADSPAPIIDGTFRDQGEYLTPS--------------------------------QPVWKAPCGSQQVIALGIQPGEN-------------------------ALSGNPKRRFSEQMG :  69
HS_18        : QVIGANSVP----GIIAPDNVHVIQPS----------NPVASGNHLQPSEVTTYPISPKVIHCDTG--------RAN--LQNLLVVNQNSAAGVQSQPIG-----YQRQYPVGTASLQT : 150
BT_18        : RVVIQNPAE----VNATQGPPTALQHP----TAVAS--LQTPPGEIQYSLGTTDLQTQPG--------------GPQNPPTCAPGPMYTSNQFQWNMPFGSSFTFDPKKFIKDEVRTLG : 156
MM_18        : QVMIQNPAG----TTGLQAGPTGLQYS-----AGMAG--VQAPPGVIQYSPEVTSVQIPPG-------------DPQNPLNTVPGPTQTSSLPQWNMSFMTFPEFNPKKFINEEVRTLG : 154
MD_8B        : SSGPPYPND----QPIPLYSNTQSIPPYP---------NIQPIPPYPNNQPQVHLYPACHSQV----------------------VLTNGNGNS------------ASKVRND-GQVFG :  93
HS_8B        : SAVPVANSV----LVVAPHNGYPVTPG-----------IMSHVPLYPNSQPQVHLVPG-NPPS----------------------LVSNVNGQP------------VQKALKE-GKTLG :  73
BT_8B        : SAGPMANSV----FVVTPPNGYTVLPG-----------GVSQVPIYP-NQSQVHVIHG-NPP--------------------------CVSQQP------------TERTLKE-GKALG :  68
MM_8A        : SPGPMANSV----YVVAPPNSYPGVPG-----------TVPQMPIYPSNQPQVHVISG-HLPG----------------------LVPAMTEPP------------AQRVLKK-GQVLG :  89
OA_8A        : S-KPANNGA----VLTPPGNGVT---------------IIQMGSNVAQGNQQVPLYSGNQFQG----------------------VVQLVNSNL------------TTGVLKEYGQVFG :  67
MM_13        : ---------------ME---------------------------------------------------------------------------------------CRNPKVSSANITVLG :  17
BT_13        : ----------------------------------------------------------------------------------------------------------------------- :   -
HS_13        : ----------------------------------------------------------------------------------------------------------------------- :   -
HS_12        : SINLENQAQ----GAQRAQPYGITSPG-----------IFASSQPGQGNIQMINPSVGT-----------------------------------------------AVMNFKEEAKALG :  92
MM_12        : PHPPQPLNF----LNVRNQVQTGQVSF---------ITSPGIFPNTQLGRENVPTVNPALGA--------------------------------------------AISNVKDAAIALG :  87
TG_MS4A15    : -----------------------------------------MQGMGSLRLGSRAVMYT------------------------------------------------AETLPKGKNRVMG :  30
AC_MS4Ax1    : PNGVNQDSQGVPGVTVTQPPGMIQYVQYE----------GPQFGSPSRPLQQNLRVSP-------------------------------------------------MVKFLNVAKTLG :  70
AC_MS4Ax2    : SGIVVHIPFDGDNAYQAGQGFPGTGTG-----------VQQLGQQFGSPGNPPEQNPPLG---------------------------------------------PLEKLAKVELKTLG :  65
DR_17A.15    : TVGAMNPST----IVIQLQPLTQTA--------------TVTSSSVPVRIQHVAGVSP---------------------------------------------LQRTHLFLRGQPKALG :  58
DR_17A.3     : TVGAMNPST----IVIQLQPLTQTA--------------TVTSSSVPVHIQHVAGVSP---------------------------------------------LQGIQLFLRGQPKALG :  58
DR_17A.2     : TVRAMNPST----IVIQLQPLTQTA--------------TGTSSSVPVRIQHVAGVSP---------------------------------------------RQGIQAFLKGQPKALG :  58
DR_17A.5     : ----MNPST----IVIQLQPPTQTA--------------TVTSSSVPVRIQRVAGVSP---------------------------------------------RQGNQAFLKGQPKALG :  52
DR_17A.1     : ----MNPST----IVIQLQPLTQTA--------------TGTSSSVPVRIQHVAGVSP---------------------------------------------LQGIQAFLKGQPKALG :  52
DR_17A.12    : TVRAMNPST----IVIQLQPLTQTA--------------TVTSSSVPVRIQHVAGVSP---------------------------------------------LQGIQAFLKGQPKALG :  58
DR_17A.4     : ----MNPST----IVIQLQPPTQTA--------------TVTSSSVPVRIQRVAGVSP---------------------------------------------LQGIQAFLRGQPKALG :  52
DR_17A.10    : TVGAMNPST----IVIQLQPLTQTA--------------TGTSSSVPVQIQHVAGVSP---------------------------------------------LQGNQAFLKGQPKALG :  58
DR_17A.6     : ----MNPST----IVIQLQQPTQTAPV-----------VTGTSSSVPVHIQHAAGVSP---------------------------------------------LQGIQAFLKGQPKALG :  55
DR_17A.11    : TVGAMNPST----IVIQLQPPTQTAPT-----------VTGTSSSVPVYIQHVAGVSP---------------------------------------------LQGIQAFLKGQPKALG :  61
DR_17A.7     : ----------------------------------------MTSSSVPVHMQHAAGVSP---------------------------------------------HQGIQLFLQGQPKVLG :  34
DR_17A.8     : TVGAMNPST----YVIQAQPPTPTAPV-----------VTATSSSVPVFIQHAAGVSP---------------------------------------------HQGIQAFLKGQPKALG :  61
DR_17A.9     : ----MNPSS----YVIQLQQPTQMAPV-----------VTATSSSVPVRIQHAAGVSP---------------------------------------------LLGIQAFLKGQPKALG :  55
DR_17A.13    : ----MNPSK----IVIQLQPQTQTA-T-----------VTGTSFSEPVYMQHEAGVS-------------------------------------------------IQAFLKGQPKALG :  50
DR_17A.16    : TVKAVDSST----VVIKLQPPTQTAPI-----------VTGTSSPVPVYIQHEAGVLT---------------------------------------------LEGIQAFLKGHTKTLV :  61
DR_17A.17    : TAGAMNPST----IVIQIQPPTQTAPI-----------VTGTSSPAPVYIRRVARVLP---------------------------------------------CQGIKAFVKGQPKALG :  61
DR_17A.14    : TVEAMSSSS----YAIQLPPRTPTAPP-----------VTGTSSSVPVHMQHVARVLS---------------------------------------------PHGVQAFLKGHPKALG :  61
DR_17B.1     : TVMPVGSST----FIIQLQPAAQTP----------------AGHANVPVSLQQLAGAPS---------------------------------------------LTELKAFLKSQPKALG :  58
DR_17B.2     : TVMPVGSST----FIIQLQPAAQTP----------------AGHANVPVSLQQLAGAPS---------------------------------------------LTELKAFLKSQPKALG :  58
DR_17C.2     : HHQTFTLTT----HFVSGE-NLKMESS-----------KIISTEKATVVIQVNPQLTQNTFVSDDGQE-----------------------------GRGSYHYMALKEFFKAQPKALG : 129
DR_17C.1     : EHQTSTTST----HFISGEIALRMESS-----------KIISTEKATVVIQINPQVTQ--LSSDDGQE-----------------------------AN-----TPLQGFFKSQPKALG : 102
OM_17C       : SVSTTTNGV----VVITHVHPAGNG-------------LGMGAPSPPTCMGRVGPVVS----------------------------------------------SVMERFREGHPKALG :  59
TN_MS4Ax3    : --------------------------------------MAAEDKRLPEAADRETGLSP---------------------------------------------------FSKMEPKTLG :  30
TN_MS4Ax2    : ---------------MEEAAAAVQNP--------------LVAITFQKNADRKK------------------------------------------------------KYLEAEPKALG :  36
SA_MS4Ax1    : -------MS----APAATQQTVVVTHL-----------YKADPSTPATTFATAPHNAY------------------------------------------------AQKFLEGEPKALG :  49
AT_MS4Ax1    : -------------MASPIGNGMFVITQY----------IPAGNAYLPEQNAQMGQTPG-----------------------------------------------FLQKFLKGEPKALG :  49
XT_MS4Ax2    : ------------MVPVQADSSSFVIIS------------RVGPQDNSADITGLSSRGPKD------------------------------------------LPVPIQKFLKGQPTVLG :  53
BT_1         : -------------MTTPRNSMVGA--------------FPAEPLKGPIAMHAAQKV--------------------------------------VPRRVPVVVGPT-QSFFMREAKALG :  53
HS_1         : -------------MTTPRNSVNGT--------------FPAEPMKGPIAMQSGPKP--------------------------------------LFRRMSSLVGPT-QSFFMRESKTLG :  53
MM_1         : --FPAEPTK----GPLAMQPAPKV-----------------NLKRTSSLVGPT------------------------------------------------------QSFFMRESKALG :  46
MD_1         : -------------MTTPRNSISGT--------------FLDDSIKGTNIIQPGQKV--------------------------------------ILRRGSQVQSPTLQGFFRRESKALG :  54
MD_15        : SAPATNGVF----VVIPPNNASGIRPG-----------PAMLPRTLCQSPGTLQFEETPLGAQPSG-------------------SVPHQRLPP------------AETFLNGEPKALG :  75
OA_15        : SAASSDGVF----VVIPPNNSSGIQPV-----------PAGLPRTLCHPPGIMQFEDQPLGMQPPR-------------------PPRSASLRP------------LETFLNGEPKALG :  75
HS_15        : AAPASNGVF----VVIPPNNASGLCPP-----------PAILPTSMCQPPGIMQFEEPPRGAQTPR-------------------ATQPPDLRP------------VETFLTGEPKVLG :  75
BT_15        : TAPSSNGVF----VVIPPSNASGLRSP-----------PAILPTSLCQPPGIMQYEEPPLGAQTPR-------------------VTQPPDLRP------------GETFLTGEPKALG :  75
MM_15        : APPASNGVF----VVIPPSNASGLRPP-----------PAILPTSMCQPPGIMQFEESQLGAQAPR-------------------ATQPPDLRP------------METFLTGEPKALG : 104
MM_5         : ---------------MDSKNVHSPL-------------FLVFPPEVTTQEYQTTE----------------------------------------LTATAYNPQNRVQKIIIRKLEILG :  51
HS_5         : ---------------MDSSTAHSPV-------------FLVFPPEITASEYESTE----------------------------------------LSATTFSTQSPLQKLFARKMKILG :  51
BT_5         : ---------------MDSRAAHSPV-------------FLVFPPDITMPEFQSGD----------------------------------------ITGTTYDSSMPFPKLLATRMKILG :  51
HS_14        : ---------------MESTSQDRRA---------------THVITIKPNETVLT-------------------------------------------AFPYRPHSSLLDFLKGEPRVLG :  46
BT_14        : ---------------MESSSEVKRS---------------THVISVQPNETVLT-------------------------------------------ALPYGPHSSLLEFLKGEPKVLG :  46
MM_2         : ---------------MDTENRSRAD-------------LALPNPQESSSAPDIELLEASP-------------------------------------AKAAPPKQTWRTFLKKELEFLG :  54
HS_2         : ---------------MDTESNRRAN------LALP--QEPSSVPAFEVLEISPQEVSSGR----------------------------------LLKSASSPPLHTWLTVLKKEQEFLG :  62
BT_2         : ---------------MEAESRSRTE-------------LALPTPQEPASASEIELAEISLQES--VL---------------------------LERPVPTAPRQTWLTVLKRELEFLG :  62
HS_6A        : -------MT----SQPVPNETIIVLPS-----------NVINFSQAEKPEPTNQGQDS------------------------------------------------LKKHLHAEIKVIG :  49
HS_6E        : -------MT----SQPISNETIIMLPS-----------NVINFSQAEKPEPTNQGQDS------------------------------------------------LKKRLQAKVKVIG :  49
MM_6D        : -------MI----PQVVTSETVTVISP-----------NGISFPQTDKPQPSHQSQDS------------------------------------------------LKKHLKAEIKVMA :  49
MM_11        : -------MI----PQVVTSETVTVISP-----------NGISFPQTDKPQPSHQSQDS------------------------------------------------LKKHLKAEIKVMA :  49
MM_6B        : -------MI----PQVVTSETVAMISP-----------NGMSLPQTDKPQPFHQWQDS------------------------------------------------LKKHLKAEIKVMA :  49
MM_6C        : -------MI----PQVVTNETITTISP-----------NGINFPQKDESQPTQQRQDS------------------------------------------------LKKHLKAEIKVIV :  49
HS_7         : -------ML----LQSQTMGVSHSFTP-----------KGITIPQREKPGHMYQNEDY------------------------------------------------LQNGLPTETTVLG :  49
MM_7         : -------MR----LQLGTKNIGWDCFP-----------KDIIIHKREKTGHTYEKEDD------------------------------------------------LLIGVPSEATVLG :  49
BT_7         : -----MPSP----LKLMTNRALDAFTP-----------KGVIISKREKPGHVYQKDD-------------------------------------------------LPKGLPKEATVLG :  50
HS_3         : ---MASHEV----DNAELGSASAHGT------------PGSETGPEELNTSVYHPING------------------------------------------------SPDYQKAKLQVLG :  52
MM_3         : ----------------------------------------MKPEETGGSVYQPLDE--------------------------------------------------SRHVQRGVLQALG :  29
MM_4B        : ---------------MQGQEQTTMAVV-----------PGVAVPSKNSVMTSQMWNEK------------------------------------------------KEKFLKGEPKVLG :  45
MM_4C        : ---------------MQGQEQTTMAVV-----------PGGAPPSENSVMKSQMWNEN------------------------------------------------KEKFLKGEPKVLG :  45
MM_9         : ---------------MQGQEQTTMAVV-----------PGGAPPSENSDMKSQMWNEN------------------------------------------------KEKFLKGEPKVLG :  45
MM_4D        : ---------------MQGLAQTTMAVV-----------PGGAPPSENSVIKSQMWNKN------------------------------------------------KEKFLKGEPKVLG :  45
HS_4E        : WCLREDRNY----AKLTFSAAMTTMQG-----------MEQTTPGAGPDVPQLGNIDVIHSYLCKGL---------------------------------------QEKFFKRKPKVLG :  71
HS_4A        : -------------------MDVPQLGN-----------MAVIHSHLWKGL--------------------------------------------------------QEKFLKGEPKVLG :  33
MM_4x        : --------M----LVIQGTEQSALEAG-----------YGAQQNGQPLYVNSHSWKRM------------------------------------------------TEKFLKGEPKILG :  48
AC_MS4Ax3    : ANGNIVIIAPNGAKTIQAGEQIPSTLMDYIR-------SGGQQNGSLSSQPQQKHCVG-----------------------------------------------AFEKIIKLEAKTLG :  73
BT_TM176B    : QNTLTVNGV----DVGSVLSQPTHIDI-----------HIHQESALAQLLKAGSSLVER-LSHR-----------------------------------------PAKARIGYGQLALG :  64
HS_TM176B    : QNTVIVNGV----AMASRPSQPTHVNV-----------HIHQESALTQLLKAGGSLKKF-LFHPGDT-------------------------------------VPSTARIGYEQLALG :  68
MM_TM176B    : QSTVTVNGV----KVASTHPQSAHISI-----------HIHQKSALEQLLGAVGSLKKF-LSWP-------------------------------------------QARIHYGQLSLG :  62
OA_TM176B    : TSTVEEDGM----EVASGDAGPAQLNI-----------HIHQESALAQLVKSGCSLFQRGFKSTRQA-------------------------------------SASRARIGRAQLALG :  76
TN_TML1.3B   : ----------------------------------------------------------------------------------------------------------------------- :   -
HS_TM176A    : --MGTADSD----EMAPEAPQHTHIDV-----------HIHQESALAKLLLTCCSALR------PRA-------------------------------------T-QARGSSRLLVASW :  58
BT_TM176A    : --METVDCG----EAAPRAPQPASIQV-----------HFHHESGLAKLLLGGCSLLQPLLLPRPRA-------------------------------------TSRALGRHRLLATSW :  65
MM_TM176A    : TDMETAVVG----KVDPEAPQPTHIDV-----------HIHQESALAKLLLAGCSLLR----IPASA-------------------------------------STQSQGSSRVLVASW :  65
DR_TMLx      : LTISQDEGL----TIVMVNSNPKSKWP-----------VLCQILGYLCYSP-VCSVSRI-----------------------------------------------MIGELANIHIGLG : 106
DR_TML1.2    : LTLAQGEGI----TVITVTSNPKSKWP-----------ILCQILGTLCYTP-VYSVSQA-----------------------------------------------VKEKLKSVYTALG :  58
DR_TML1.3A   : SWCKRATPA----KLVEDTKSETQRVG-----------ICTQSYSYFKSCNMTFAGTQD-----------------------------------------------LKDKLTGTHKALG : 176
TN_TML1.3A   : VTVANSEGV----TVLTLTADPNSACP-----------PLCQILNGLCCSPRCCTASQQ-----------------------------------------------LKENQGTSQSLLG :  59
DR_TML1.3B   : LSVCQDDGT----IVITISTNPKSKWP-----------ILCQILGTLCYSP-VCSESQN-----------------------------------------------SKTKMMNNYTALG :  58
BT_12        : --------------------------------------------------------------------------------------------------------MTRRFPLRTEARALG :  15
TN_MS4Ax1    : ----------------------------------------------------------------------------------------------------------------------- :   -
XT_MS4Ax1    : ------------------------------------------------------------------------------------------------------------------MVVLG :   5
TN_MS4Ax4    : ---------------MS--------------------SEEVPADGPRSHRLFPWFDP---------------------------------------------------ESAAVVTILLG :  33
SP_out       : VAYPQGQPV----AYPQGQPVVYAQGQ----------PGVVYPPGQQVVITQGAATHVYQTGSSSGS-----------------------------------CNSTSEHYNQKAGFATG : 130
```

```
              40         *        260         *        280         *        300         *        320         *        340         *
MM_10       : GFHIAIAFAHLAFGGYLI----------STVKN-----LHLVVLK---CWYPLWGTVSFLVAGMAAMTTV----TFPKTSLKVLCVIANVIS-LFCALAGFFVIAKDLFLEGPFP---- : 150
HS_10       : AFHITIALLHLVFGGYLA----------SIVKN-----LHLVVLK---SWYPFWGAASFLISGILAITMK----TFSKTYLKMLCLMTNLIS-LFCVLSGLFVISKDLFLESPFE---- : 153
BT_10       : AVHVVIALVYLFIGGYLV----------AAVQN-----LHLVVQK---CWYPFWGAASFLISGILAITME----SFQKNYLKM-CLVANSIS-FLCVLAGLFVIAKDLFLETPFE---- : 157
OA_10       : IFHFMSAIIHFSFGIWLM--------------AISQNLHLVALKS----WYPIWGSLSYFLSGGLSAIVE----KIPKRGMKPCCLVMNIFS-IFCALSGVFVFIKDLFLENVFP---- : 161
HS_18       : AIQILIGLTHIFSAINPV------------LYYYPF----VTW----LSGYPLWGGLSYIVSGSLSVWAA---KDP-SPCVVNSSISFNIIS-ALFAFAGIFIIITDLSLYY------- : 237
BT_18       : AIQILIGLTHIFTAINPS------------LYRQYS----YSA----ISGYLVWGGIFFIISGSLSVEAE---KDR-SSCMVHGSVGMNVVS-AIVSLAGVLLLLVDLIRNP------- : 243
MM_18       : AIQILIGLFHIFSAVNPQ----------LYNTQF----FVVPLLG--VSGYLIWGGLSFIISGSLSVLAE---KNS-NSCVVNSSIGMNVVS-SIFSLTGIIIIIVQLSIYPSEI---- : 248
MD_8B       : AIQILIGLLHIGFGSVLV-------------TSVIGGHISISFWG----GYPYWGGISFITSGTLTVLAQ---KPPITSCKTNGSLGANIVS-AIFSAVGVILLITDFSINWGRY---- : 187
HS_8B       : AIQIIIGLAHIGLGSIMA-------------TVLVGEYLSISFYG----GFPFWGGLWFIISGSLSVAAE---NQPYSYCLLSGSLGLNIVS-AICSAVGVILFITDLSIPHP-Y---- : 166
BT_8B       : ATQILIGLIHLGLGSVMG-------------TVLMGNYTAVSFYG----GFPFWGGIWFIISGSLSVLAE---KHSRSSCLLNSSVGFNIVS-AIFSMVGITLFIVELIINST-Y---- : 161
MM_8A       : AIQILIGLVHIGLGSIMI-------------TNLFSHYTPVYLYG----GFPFWGGIWVIISGSLSVAGE---TQPNSPCLLNGSVGLNIFS-AICSAVGIMLFITDISISSG-Y---- : 182
OA_8A       : AIQIMIGLIHIIFGVALG-------------TEVTVRYLSLAFYG----AYPFWGGLSFIISGTLSVSSQ---RNP-TICLVNGSVGMNILS-AIFSVTGILLLIAELCLNHP------ : 158
MM_13       : VIQIMIGIYHVLMWYFLLLLYMGQI------KGVFGTYEPVTYKM----GTSLWG-FAFVISGAFTVKAA---KYQ-SRHMILCTMSLNILC-IIITIVAASLTIVELSHFRS------ : 114
BT_13       : ----MTGIFCIFMWYLLLILYMGQI------KGVFGTYEPITYKT----GCSLWG-IFFIISGISIIRAT---WYP-SQRQLTCAMLENILC-MILAIISMILTIVELSTFKS------ :  93
HS_13       : ----MIGIFHVFMWYFLLVLYMGQI------KGAFGTYEPVTYKT----GCTLWG-IFFIIAGVFLIRVT---KYP-TRSGIISTLIINIIC-IITTITAVTLTIIELSHFNS------ :  93
HS_12       : VIQIMVGLMHIGFGIVLC----------LISFSFREVLGFASTAV--IGGYPFWGGLSFIISGSLSVSAS---KEL-SRCLVKGSLGMNIVS-SILAFIGVILLLVDMCINGVAG---- : 190
MM_12       : VIQITIGLMHIGFGVVLG----------LLSTSYNMTWAFSSVAF--IGGYPFWGGVFFIASGSLSISAF---KEF-SHCLMKSTLIINISS-TIFAFVGVILFLCDLNINGYYY---- : 185
TG_MS4A15   : TIQIMTGFLHIGFGIVLT--------------TLTNVYTSVFVIG----EIPFLGGVSFIISGCLSIGAE----KSPTECAVKGSQTMNVIS-AIFALLGIVAFIVDLNLNGLYR---- : 122
AC_MS4Ax1   : AVQIFIGLIHIGLGAVLAAIWG------------FPCIISIAVVG----GYVFWGALFFIISGSLSIAAE----KQATTGLVKSSVGMNIVS-AIMALTGSLLLLVDLLMVNLSS---- : 164
AC_MS4Ax2   : AVQIMIGLLNIGLVTILFD------------------------------WLVLCGGLFFIVSGSLSVAVE----KRLTRGLVRCSVGMNLTS-AIISLIGIILCIIGLAVVNHYLSS-- : 147
DR_17A.15   : TVQIMIGVLTFLFGILFI----------G------YEPFFFINTG-----VSFWGSLIYIAAGSLSIAAENKINSPPALCLVRASLGMNIFS-AIAAGISIIVHSMDLGFGSIYH---- : 151
DR_17A.3    : TVQIMIGVLTLLLVSVCS----------G------FRNYFFLNT-------FIWGSLIYITAGSLSIAAENKINSPSALCLVRASLVMNIFS-AIAAGIAIFVLSVFLALGSIHS---- : 149
DR_17A.2    : TVQIMIGVLTFLLGILCT----------I------YGNYIFFFTG-----VSYVASLIYITTGLLSIAAENKINSPSALCLVRASLGMNIFS-AIAAVFSIIMLSLGLAVGPTNR---- : 151
DR_17A.5    : TVQIMIGVVTLLLEIVCT----------S------YGYYLFLFTG-----ASYWGSLIYIIAGSLSIAAENKINSPTGLCLVRASLGMNIFS-AIAAGISIIVLPIGLAFGPFYS---- : 145
DR_17A.1    : TVQIMIGVLTFLLGIVCT----------S------YGYYVFLFTG-----ASYWGSLIYITAGSLSIAAENKINSPSALCLVRASLGMNIFS-AIAAGIAIIVLSVNLAFGSIYH---- : 145
DR_17A.12   : TVQIMVGVLTFLLGIASI----------H------YGYYVFFHTG-----ASYWGSLIYIIAGWLSIAAKNKIDSPAGLCLVRASLVMNIFS-AIAAGISIIEHSLNLVFVSSGP---- : 151
DR_17A.4    : TVQIMIGVLTLLFGIASI----------G------YEMFFFINTG-----VSYWASLIYITAGSLSIAAETNIISPAGLCLVRASLGMNIFS-AIAAGISIIVISVNLAFAPVFG---- : 145
DR_17A.10   : TVQILIGLWILLLGIAS---------------------TAIFYTG-----VPYWASLIYIIAGSLSIAAENKIFSPAGLCLVRASLGMNIFS-AIAAGISIIVLSLSLAFGPTR----- : 145
DR_17A.6    : AVQIIIGLWTFLLGIVS---------------------TAFAFSG-----IPYGASLIYITAGSLSIAAETKINSPAGLCLVRASLGMNIFS-AIAAGISIIMLSVQLALGSIH----- : 142
DR_17A.11   : TVQIIIGLLTFMLGIVST----------V------YVDSSFVFSG-----VSYWGSLIYIIAGSLSIAADSKINLPAGLCLVRASLGMNIFS-AIAAGIAIIMLSVDLALGPFSP---- : 154
DR_17A.7    : AVQIMIGAWTFLLGIVT---------------------FAYAAGG-----VSYGASVIYIIAGSLSIAAEIKINSPDALCLVRASLGMNIFS-AIAAGSSIIVLSQAMAFARIVT---- : 122
DR_17A.8    : TVQIMIGAWTFLFGIVS---------------------IVFAVGG-----VSYVASPIYITAGSLFIAAENQINSPAGLCLVRASLGMNIFS-AIAAGISIIVLSQGLAFDRINS---- : 149
DR_17A.9    : TVQIMIGVVTFLFGIVS---------------------TLFAAGG-----VSYVASLIYITAGSLSIAAENKINSPSALCLVRASLGMNIFS-AIAAGSSIIMLSLGLAFGRIIS---- : 143
DR_17A.13   : TVQIIIGLLILLLEIVFT----------V------FTHSTFAFSG-----VPYVGSLIYITAGSLSIAAENKIDSPAGLCLVRASLGMNIFS-AIATGIAIIMLSLGLTIGP-YT---- : 142
DR_17A.16   : NVQIMIGVLTFLFGIVLT----------V------YAESVFVFSG-----IPYWGSLIYIAAGSLSIAAENKHDSPTSLCLVKGSLGMNIFS-AVTAAIAFILLSVDLGNGPLYL---- : 154
DR_17A.17   : TVQIMIGLFTLLLGIAST----------I------DAASIFVFSG-----APYWGSVLYITAGSLAIDAENKRNSPTNLCLVKGSLGMNIFS-AIVAGTAIVIISMDMLTGQFYG---- : 154
DR_17A.14   : TVQIMIGLLSLLLGIAST----------V------YADSAFVFTG-----VPFWASAIYIIAGSLAVAAENQINSPAALCLMNGSLGMNVFS-AIAAGIAIIFLSLDLVNGPNCR---- : 154
DR_17B.1    : TVQIMIGLLTLLFGIVSA----------I------YVNSPFASLG-----CPFWGSVIYISTGSLCIAAENRLNSPSSLCLVKGSLGMNIIS-AIIAGFSIILLVVDLSVNSMYS---- : 151
DR_17B.2    : TVQIMIGLLTLLFGIVSV----------I------YVNSPFAGLG-----CPFWGSVIYISTGSLCIAAENRLNSPSSLCLVKGSLGMNIVS-AIIAGFSIILLAMDLTVNSMYS---- : 151
DR_17C.2    : TVQIMTGVIVFLFGIVLT----------IDFDYRYHDPHLSVASG-----ITYWGSLVYISAGSLSVAAQNKLH----ACVVKASLIMNVLS-AITAAAAITLMSIELFAGDNYH---- : 224
DR_17C.1    : TVQIMIGVIVFLLGTVLT----------FNVRY---QPAIVVFSG-----ITYWGSFIYISAGSLSVVAQNHLH----LCVVKASLGMNVFS-TVTSGIAIILLGIDFGLLISPS---- : 194
OM_17C      : TVQIMFGAMTFLFGIVMA----------VG------ELSLGVVSG-----IFVWGSIIYIIAGSLTIAAEKSLN----KCLVKASLGINVVA-TVTAGIGTVLYSVDA--TGVFY---- : 146
TN_MS4Ax3   : AIQIVIAALILCLSASVL-------------------QLHEVHFTG--DVALFLIVVLQVTLSGSVLVHSG----RKPSLFWVKCVLVLHLI-SAAFATAALGLMSKHLPYRQDSY---- : 120
TN_MS4Ax2   : ITQIVLSAFQIVCVSVFL--------------------GQGLSSWG--IDVAFFISSLLVIIAGSVAIAAQN-----LHLPTLRACLGMQIVA-CGATLFNIISSMAKYEIMPSYC---- : 124
SA_MS4Ax1   : VTQIMIGLVQLTFTIPIL----------LTVVSDSIG-----------LGSPFLMGVAFIISGALSIAAE----NNPHIRLLQACLATNIIS-SFLAVYGAITYFIDIMLTPSCE---- : 138
AT_MS4Ax1   : VVQIMIGIINIFLGGCLCT----------------VEYPLVAVIS----GVVFWGGILFIISGSLSISAE----KKLTDCLVKGSLAMNVIS-AVASGIELIICIIDLVISRYAYR--- : 140
XT_MS4Ax2   : VIQLFTGITQIAAGIVLC---------------IAMASRYHVISG---SGVPFWTGVMYMISGALSVAAS----SKPTLGKMKSSMALNIVS-CVCAAIAVIIYIVIILDIRYYRAHQA : 149
BT_1        : AVQIMNGLFHIALGS-LM----------LIHMDVYM----PICVT---LWYPLWGGIMFIISGSLLAAA----EKNSTQSMLTGRLIMNSLS-LFAAISGIIFLIMDIFNMTISH---- : 145
HS_1        : AVQIMNGLFHIALGG-LL----------MIPAGIYA----PICVT---VWYPLWGGIMYIISGSLLAAT----EKNSRKCLVKGKMIMNSLS-LFAAISGMILSIMDILNIKISH---- : 145
MM_1        : AVQIMNGLFHITLGG-LL----------MIPTGVFA----PICLS---VWYPLWGGIMYIISGSLLAAAA---EKTSRKSLVKAKVIMSSLS-LFAAISGIILSIMDILNMTLSH---- : 139
MD_1        : AVQIMIGLFHFGLGGILL----------MVPVGPYA----PVCVA---VWYPFWGGIIYITSGSFLVAS----EKTSRSCLVNTKLGMNAIS-LFSSISGLILLIMDIFNLTVSH---- : 147
MD_15       : TVQILIGLIYISFGSVLL----------MFRRGY-----IGMLFI--DGGIPFWGGVCFIISGSLSVATE---KYH-SSCLVKISLGMNIIN-IIASFAATAILLMDFGVTNWD----- : 167
OA_15       : TVQILIGLIYISFGSVLL----------MFRRGH-----MGMFFI--DGGIPFWGGLCFIISGSLSVAAE---KNH-TNCLVKSSLGMNLVS-TIASFAGTAILLMDFGVTNWD----- : 167
HS_15       : TVQILIGLIHLGFGSVLL----------MVRRGH-----VGIFFI--EGGVPFWGGACFIISGSLSVAAE---KNH-TSCLVRSSLGTNILS-VMAAFAGTAILLMDFGVTNRD----- : 167
BT_15       : TVQILIGLIHLGFGGVLL----------MVRRSH-----VGMLFI--EGGVPFWGGACFIISGSLSVAAE---KNH-TSCLVRSSLGTNILS-AMAAFAGTAILLMDFGVTNWD----- : 167
MM_15       : TVQILIGLIHLGFGSVLL----------MVRRGH-----LGMLFI--EGGVPFWGGACFIISGSLSVAAE---RNH-TSCLLKSSLGTNILS-AMAAFAGTAILLMDFGVTNWD----- : 196
MM_5        : TTQILFGIMNFSFGVIFL---------FTLVNPYPRF--PFIFI----SGYPFWGSVLFINSGAFLIAL----KRKTTDTLIKMSRAMNLLS-ALGAAAGIILLIFGFLLDGEFICG-- : 148
HS_5        : TIQILFGIMTFSFGVIFL---------FTLLKPYPRF--PFIFL----SGYPFWGSVLFINSGAFLIAV----KRKTTETLIILSRIMNFLS-ALGAIAGIILLTFGFILDQNYICG-- : 148
BT_5        : AVQILLGAMNFSFGTVLL---------FTLEDPYPRF--PFIFI----SGYPFWSSILFVNSGAFLVAL----ERKTTETLVTLSRIMNSLS-AVAAAAGIILLIIGFLIDRDFLCG-- : 148
HS_14       : ATQILLALIIVGFGTIFA------------LNYIGFSQRLPLVVL---TGYPFWGALIFILTG-YLTVTD-----KKSKLLGQGVTGMNVIS-SLVAITGITFTILSYRHQDKYC---- : 139
BT_14       : ASQVLLALIIAGIGAIFA------------LNYFNFAQRFPLVFL---TGYPFWGAFIFTVTG-YLTGAH-----SNDKCVGQGVTGLNVIS-SVVAVAGIILTIISYRYQHKYC---- : 139
MM_2        : ATQILVGLICLCFGTIVC----------SVLYVSDFDEEVLLLYK---LGYPFWGAVLFVLSG-FLSIIS---ERKNTLYLVRGSLGANIVS-SIAAGTGIAMLILNLTNNFAYM---- : 151
HS_2        : VTQILTAMICLCFGTVVC----------SVLDISHIEGDIFSSFK---AGYPFWGAIFFSISG-MLSIIS---ERRNATYLVRGSLGANTAS-SIAGGTGITILIINLKKSLAYI---- : 159
BT_2        : VTQILIGLIYLYFGIIVS----------AKINDSEFTEKFFSSFK---AGYPFWGALFFAISG-ISSIMS---EKKPSAYLIRGCLGANAVS-GVAAGTGVGILISNLKQSATYI---- : 159
HS_6A       : TIQILCGMMVLSLGIILA----------SASFSPNFT-QVTSTLL--NSAYPFIGPFFFIISGSLSIATE----KRLTKLLVHSSLVGSILS-ALSALVGFIILSVKQATLNPAS---- : 146
HS_6E       : ------------------------------------------------VHSSLAGSILSALSALVG-------FILLSVNPAALNPAS--LQCKLDEKDIPTRLLL--------- :  98
MM_6D       : AIQIMCAVMVLSLGIILA----------SVPSNLHFT-SVFSILL--ESGYPFVGALFFAISGILSIVTE----KKMTKPLVHSSLALSILS-VLSALTGIAILSVSLAALEPAL---- : 146
MM_11       : AIQIMCAVMVLSLGIILA----------SGPSNLHFT-SVVSILL--ESGYPFVGALVFAISGILAIGTE----KKMTKPLGHSSLALSILR-VLSALTGIAILSVRLAALEPAL---- : 146
MM_6B       : AIQIMCAVMVLSLGIILA----------SVPSNLHFT-SVFSVLL--KSGYPFIGALFFIVSGILSIVTE----TKSTKILVDSSLTLNILS-VSFAFMGIIIISVSLAGLHPAS---- : 146
MM_6C       : AIQIMCAVTVLALGIILA----------SVPPVPYFN-SVFSVLL--KSGYPFIGALFFIASGILSIITE----RKSTKPLVDASLTLNILS-VSFAFVGIIIISVSLAGLHPAS---- : 146
HS_7        : TVQILCCLLISSLGAILV----------FAPYPSHFN-PAISTTL--MSGYPFLGALCFGITGSLSIISG----KQSTKPFDLSSLTSNAVS-SVTAGAGLFLLADSMVALRTAS---- : 146
MM_7        : TIQLLCALILASFGGILV----------SA---SYFN-PEVSTTL--ISGYLFIGSLCFAIAGILSIISE----KISTKPFALSSLASNVAS-SVVAVIGLFLFTYCLIALGSAF---- : 143
BT_7        : IIQILCFLVISSLGVILV----------SVPNSSQLS-PAISSIL--MTGYPFFGALCFAITGTVSIISG----KKSNKPFAINSLIFSAVS-SVAAGVGLILLAGSLVALRTAS---- : 147
HS_3        : AIQILNAAMILALGVFLG----------SLQYPYHFQKHFFFFTF--YTGYPIWGAVFFCSSGTLSVVAG----IKPTRTWIQNSFGMNIAS-ATIALVGTAFLSLNIAVNIQSL---- : 150
MM_3        : AIQILNGILILALGIFLV---------CLQHVSHHFRHFFFFTF---YTGYPLWGAVFFISSGSLTVAAG----RNPTRMLMQNSFGINIAS-TTIAFVGTVFLSVHLAFNTQAF---- : 127
MM_4B       : VLQVMIAIINLSLGIIIL----------TTLFS------ELPTSV--MLMVPIWGSIMFIVSGSLSIAAG----VTPTKCLIVASLTLNTIT-SVLAATASIMGVVSVAVGSQFP---- : 137
MM_4C       : VVQVMIALINLSFGIIIL----------ANLSS------EPLISV--VLMAPIWGPIMFIVSGSLSIAAG----VKPTKSLIISSLTLNTIT-SVLAATASIMGVVSVAVGSQFP---- : 137
MM_9        : VVQVMIALINLSFGIIIL----------ANLSS------EPLIYA--VLMAPSWGPIMLIGSGSLAIAAG----VKPTKSLIISRLTLNTIT-SALAATASIMGVVSVAVGSQVP---- : 137
MM_4D       : AIQVMIAFINFSLGIIII----------LNRVS------ERFMSV--LLLAPFWGSIMFIFSGSLSIAAG----VKPTKAMIISSLSVNTIS-SVLAVAASIIGVISVISGVFRQ---- : 137
HS_4E       : VVRILIALMSLSMGIIMM----------CVAFSSY---EEHPIFV--YVAYTIWGSVMFIISVSLSVAAG----IRTTKGLVGGSLGKNITS-SVLAISGILINAISLTFYSFRY---- : 166
HS_4A       : VVQILTALMSLSMGITMM----------CMASNTY---GSNPISV--YIGYTIWGSVMFIISGSLSIAAG----IRTTKGLVRGSLGMNITS-SVLAASGILINTFSLAFYSFHH---- : 128
MM_4x       : IVQIVIAIMNLSIGIMMI----------IATVSTG---EIPPSSV--YIGYPIWGSLMFIISGSFSIVAG----RRTTKGLVRSSLGLNITS-SVFAFSGIVISSLSPGIYSFHV---- : 143
AC_MS4Ax3   : VLQIMIGLIHFGFGIISVIN---------------AVNHFLTALS---TIFPVYSSLIFIASGSLSVSAE----KHWNTNLKNTSVAINILS-AIMASLGIIWYIFDIAQIVRLP---- : 165
BT_TM176B   : VTQLLLGAISCALGGLLYLGP----------------WIQLRASG-----CALWAGFVAIAAGVGAIVHE----KRRGKLSGCVSGLLTLAG-IATAVAAVVFCVNSFIWQYDG----- : 152
HS_TM176B   : VTQILLGVVSCVLGVCLSLGP----------------WTVLRASG-----CAFWAGSVVIAAGAGAIVHE----KHPGKLAGYISSLLTLTG-FATAMAAVVLCVNSFIWQTEP----- : 156
MM_TM176B   : VTQILLGLVSCALGVCLYFGP----------------WTELCAFG-----CAFWSGSVAILAGVGTIVHE----KRQGKLSGQVSCLLLLAC-IATAAAATVLGVNSLIRQTSVPYYVE : 155
OA_TM176B   : VTQVVLGTVSGALGVLLYLGP----------------WIMLSSTG-----CAFWPGAVAIAAGAGAIVHE----KRRSRCWGGVSVLMTLLS-ASAALAALILCINSFVWQPDG----- : 164
TN_TML1.3B  : ----------------------------------------MRDSGFP-----FWFGGMLILIGASCILSE----RRPSPNRVTLNVTLNIVG-FALAVAAIVLYINSVADTWLE----- :  64
HS_TM176A   : VMQIVLGILSAVLGGFFYIRD----------------YTLLVTSG-----AAIWTGAVAVLAGAAAFIYE----KRGGTYWALLRTLLTLAA-FSTAIAALKLWNEDFRYGYS------ : 145
BT_TM176A   : VMQIVLGLLSGVLGGFLYIFS----------------STTLRNSG-----APIWTGAVAVLAGAVAFIYE----KRGGIYWALLRTLLALAA-FSTATAATIIGAGRFYEYHFI----- : 153
MM_TM176A   : VVQTVLGALSVVLGGTLYIGH----------------YLAMYSEG-----APFWTGIVAMLAGAVAFLHK----KRGGTCWALMRTLLVLAS-FCTAVAAIVIGSRELNFYWYF----- : 153
DR_TMLx     : IVQMIIGVLNIAMGTL------------LMCLGYYE--NMFYMGQ-----GPFWIGGVVLGIGVVCILVA----KFPSSCLLIIGMLLNMIS-AGLAIAAIVLYAKDLSYGHDQ----- : 196
DR_TML1.2   : TMQIIYGVLCIVLGVL------------IDRLWYWN--NLTGCD------AVFWFGGTAIVFGIVTILAA----WFPSSCLLAFTLLLNVVS-GALAITTVVLCSIDLVRGNYL----- : 147
DR_TML1.3A  : IVQIVVGVMNITVGFFGFG-------------------------------SSFLIGGVFLVIGIMCVLAV----KFPSQRLLVILVFLNIVS-AAMAISAVVLYSLDLAMGNNF----- : 254
TN_TML1.3A  : TLHILAGLLNILLSVILCAGG---------RYPWPIRESYFP----------FWIGGMYILIGAMCVWSE----RRPSPCLVILNVHLNIVG-LAFAVAAIVLYSQSLADLYIR----- : 149
DR_TML1.3B  : IVQVIVGILSLGTGNQ------------------------FLR---------FWLGSVFLMVGIVTVVAV----RFPSDFLLLTAAVLNTVS-ALLAMMGLAFSASDMAS--------- : 130
BT_12       : AVQIVLALFHYTLGFLCA-------VLFLGEEDVKVTGSVPVLLT---LGYIIWSSPFFLISGCVAIIAQ----KKPTRYQLTSAIVMNIFSACFSAFGTIILCIACLSYVAEM----- : 115
TN_MS4Ax1   : -----------------------------MGRPLLTEDVMYFG---SYIPFWQGFLFLVCGSLSIYTE----AHPSKKLVIVCLSMYIVT-VLGIVVSFFYRAINLNFYGWPI---- :  76
XT_MS4Ax1   : ILQIACAGISIVSGFMDG---------------AFRRESTLGRTR-----APLWAGLIMTIPGVLALFSS----QKKNPVLVNALIAASVFS-CFTTLIVIVYASLTLGYGEDDD---- :  95
TN_MS4Ax4   : LFQVLLSALLINTEGPVP--------------------KLFV--------LPLLLGILIVAGGSLTIANE----RNPSRLLLQQCVTSNVAG-LVGTLLALCLYGYSLSAVPKTV---- : 115
SP_out      : IVHLIVAILSVILGGVAY-------------------VFPVYQVG-----YGIWSAFIFYIPTGILGVAS----KKKNSCVIIAYMVMAILS-TFHAFGMLAYESTVAAVLTYVE---- : 216
```

```
                360         *       380         *       400         *       420         *       440         *       460         *
MM_10       : -----WPIWRPYP----------------------------------------------------------------------------------------------------------- : 158
HS_10       : -----SPIWRMYP----------------------------------------------------------------------------------------------------------- : 161
BT_10       : -----FPIWKPYP----------------------------------------------------------------------------------------------------------- : 165
OA_10       : ------LWTLYP------------------------------------------------------------------------------------------------------------ : 167
HS_18       : ------------------------------------------------------------------------------------------------------------------------ :   -
BT_18       : ------------------------------------------------------------------------------------------------------------------------ :   -
MM_18       : ------SP---------------------------------------------------------------------------------------------------------------- : 250
MD_8B       : ------SYFDS------------------------------------------------------------------------------------------------------------- : 192
HS_8B       : ------AYP--------------------------------------------------------------------------------------------------------------- : 169
BT_8B       : ------IYP--------------------------------------------------------------------------------------------------------------- : 164
MM_8A       : ------IYP--------------------------------------------------------------------------------------------------------------- : 185
OA_8A       : ------------------------------------------------------------------------------------------------------------------------ :   -
MM_13       : ------------------------------------------------------------------------------------------------------------------------ :   -
BT_13       : ------------------------------------------------------------------------------------------------------------------------ :   -
HS_13       : ------------------------------------------------------------------------------------------------------------------------ :   -
HS_12       : ------QDY--------------------------------------------------------------------------------------------------------------- : 193
MM_12       : ------QDY--------------------------------------------------------------------------------------------------------------- : 188
TG_MS4A15   : ------------------------------------------------------------------------------------------------------------------------ :   -
AC_MS4Ax1   : ------YSYDHDHYY--------------------------------------------------------------------------------------------------------- : 173
AC_MS4Ax2   : -----DPPDYSHHN---------------------------------------------------------------------------------------------------------- : 155
DR_17A.15   : ------YWAKPY------------------------------------------------------------------------------------------------------------ : 157
DR_17A.3    : ------NC-TGS------------------------------------------------------------------------------------------------------------ : 154
DR_17A.2    : ------YC-RDF------------------------------------------------------------------------------------------------------------ : 156
DR_17A.5    : ------------------------------------------------------------------------------------------------------------------------ :   -
DR_17A.1    : ------SC-RDS------------------------------------------------------------------------------------------------------------ : 150
DR_17A.12   : ------SC---------------------------------------------------------------------------------------------------------------- : 153
DR_17A.4    : ------FCGHPD------------------------------------------------------------------------------------------------------------ : 151
DR_17A.10   : ------SYCTDT------------------------------------------------------------------------------------------------------------ : 151
DR_17A.6    : ------YYCRDS------------------------------------------------------------------------------------------------------------ : 148
DR_17A.11   : ------RYCGDSY----------------------------------------------------------------------------------------------------------- : 161
DR_17A.7    : ------RCRDAH------------------------------------------------------------------------------------------------------------ : 128
DR_17A.8    : ------HCIDFR------------------------------------------------------------------------------------------------------------ : 155
DR_17A.9    : ------QCTDFH------------------------------------------------------------------------------------------------------------ : 149
DR_17A.13   : ------YCRLYN------------------------------------------------------------------------------------------------------------ : 148
DR_17A.16   : ------YCSGY------------------------------------------------------------------------------------------------------------- : 159
DR_17A.17   : ------YCSDY------------------------------------------------------------------------------------------------------------- : 159
DR_17A.14   : ------YCTWHYD----------------------------------------------------------------------------------------------------------- : 161
DR_17B.1    : ------FFDYSVH----------------------------------------------------------------------------------------------------------- : 158
DR_17B.2    : ------FWDYSGH----------------------------------------------------------------------------------------------------------- : 158
DR_17C.2    : ------CNYS-------------------------------------------------------------------------------------------------------------- : 228
DR_17C.1    : ------CDYSSYY----------------------------------------------------------------------------------------------------------- : 201
OM_17C      : ------SCYNYYG----------------------------------------------------------------------------------------------------------- : 153
TN_MS4Ax3   : ---HCEHCRRLEQHAVQHCFRKCTG----------------------------------------------------------------------------------------------- : 142
TN_MS4Ax2   : ------WYSSYEN----------------------------------------------------------------------------------------------------------- : 131
SA_MS4Ax1   : ------TAS--------------------------------------------------------------------------------------------------------------- : 141
AT_MS4Ax1   : ------SDCSYSN----------------------------------------------------------------------------------------------------------- : 147
XT_MS4Ax2   : MCVCYELNSTCKG----------------------------------------------------------------------------------------------------------- : 162
BT_1        : ------FFK--------------------------------------------------------------------------------MENLNLIKTQVPYININNCERA--------- : 170
HS_1        : ------FLK--------------------------------------------------------------------------------MESLNFIRAHTPYINIYNCEPA--------- : 170
MM_1        : ------FLK--------------------------------------------------------------------------------MRRLELIQTSKPYVDIYDCEPS--------- : 164
MD_1        : ------FLK--------------------------------------------------------------------------------LKSLNLGQTATPYVNIYNCEQN--------- : 172
MD_15       : ------------------------------------------------------------------------------------------------------------------------ :   -
OA_15       : ------------------------------------------------------------------------------------------------------------------------ :   -
HS_15       : ------------------------------------------------------------------------------------------------------------------------ :   -
BT_15       : ------------------------------------------------------------------------------------------------------------------------ :   -
MM_15       : ------------------------------------------------------------------------------------------------------------------------ :   -
MM_5        : ------YSPD-------------------------------------------------------------------------------------------------------------- : 152
HS_5        : ------YSHQ-------------------------------------------------------------------------------------------------------------- : 152
BT_5        : ------YSGE-------------------------------------------------------------------------------------------------------------- : 152
HS_14       : ------QMPSFEEICVFSRTLF----------------------------------IVLFFLPSDVTQNSEQPAPEENDQLQFVLQEEFSSDDSTTNAQSVIFGGYAFFKLTLSRSPLVS : 219
BT_14       : ------HGPSLEGICVIGRVLYNGILSVLLIISIAELSIAVTIASFRSNCWTNSNEIVFFLPSDVTQESELSVPDENAVIQFELQEESTSADSTTNIKPVFFGGYSFFKLRVTKNQFTF : 252
MM_2        : ------N------------------------------------------------------------------------------------------------------------------ : 152
HS_2        : ------HIH--------------------------------------------------------------------------------------------------------------- : 162
BT_2        : ------Y------------------------------------------------------------------------------------------------------------------ : 160
HS_6A       : ------LQCELDKNNIPTRSYV-------------------------------------------------------------------------------------------------- : 162
HS_6E       : ------------------------------------------------------------------------------------------------------------------------ :   -
MM_6D       : ------QQCKLAFTQLDT-TQD-------------------------------------------------------------------------------------------------- : 161
MM_11       : ------------------------------------------------------------------------------------------------------------------------ :   -
MM_6B       : ------EQCLQSKELRPT-EYH-------------------------------------------------------------------------------------------------- : 161
MM_6C       : ------EQCKQSKELSLI-EHD-------------------------------------------------------------------------------------------------- : 161
HS_7        : ------QHCGSEMDYLSSLPYS-------------------------------------------------------------------------------------------------- : 162
MM_7        : ------PHCNSEKKFLSLLSYL-------------------------------------------------------------------------------------------------- : 159
BT_7        : ------QQCDSGRDYLSSMPYS-------------------------------------------------------------------------------------------------- : 163
HS_3        : ------RSCHSSSE---------------------------------------------------------------------------------------------------------- : 158
MM_3        : ------KGCQSSP----------------------------------------------------------------------------------------------------------- : 134
MM_4B       : ------FRYNY------------------------------------------------------------------------------------------------------------- : 142
MM_4C       : ------FRYNY------------------------------------------------------------------------------------------------------------- : 142
MM_9        : ------IRDNY------------------------------------------------------------------------------------------------------------- : 142
MM_4D       : ------FRSQP------------------------------------------------------------------------------------------------------------- : 142
HS_4E       : ------HYCNHDQL---------------------------------------------------------------------------------------------------------- : 174
HS_4A       : ------PYCNYYGN---------------------------------------------------------------------------------------------------------- : 136
MM_4x       : ------YYCTYRGS---------------------------------------------------------------------------------------------------------- : 151
AC_MS4Ax3   : ------KQLISSS----------------------------------------------------------------------------------------------------------- : 172
BT_TM176B   : -FYDISSVCDSLVPVTPTSW---------------------------------------------------------------------------------------------------- : 171
HS_TM176B   : -FLYIDTVCDRSDPVFPTTG---------------------------------------------------------------------------------------------------- : 175
MM_TM176B   : IFSTCNPLQSSMD-PG-------------------------------------------------------------------------------------------------------- : 170
OA_TM176B   : -FYYMDSVCESPSPTSWTPR---------------------------------------------------------------------------------------------------- : 183
TN_TML1.3B  : ------WMCDHD------------------------------------------------------------------------------------------------------------ :  70
HS_TM176A   : ---YYNSACR-ISSSSDWN----------------------------------------------------------------------------------------------------- : 160
BT_TM176A   : ---FYKGICN-VSPS--WRP---------------------------------------------------------------------------------------------------- : 167
MM_TM176A   : ---LGDDVCQ-RDSSYGWST---------------------------------------------------------------------------------------------------- : 169
DR_TMLx     : ------YCRSYYYYQS-------------------------------------------------------------------------------------------------------- : 206
DR_TML1.2   : ------YCDDYY------------------------------------------------------------------------------------------------------------ : 153
DR_TML1.3A  : ------SCYENYG----------------------------------------------------------------------------------------------------------- : 261
TN_TML1.3A  : ------WICERDY----------------------------------------------------------------------------------------------------------- : 156
DR_TML1.3B  : ------------------------------------------------------------------------------------------------------------------------ :   -
BT_12       : ------EDYVWS------------------------------------------------------------------------------------------------------------ : 121
TN_MS4Ax1   : ------RHLSFS------------------------------------------------------------------------------------------------------------ :  82
XT_MS4Ax1   : -----DIFSSPP------------------------------------------------------------------------------------------------------------ : 102
TN_MS4Ax4   : -----CPLIPTR------------------------------------------------------------------------------------------------------------ : 122
SP_out      : -----SCYFRYTG----------------------------------------------------------------------------------------------------------- : 224
```

```
                480         *       500         *       520         *       540         *       560         *       580         *
MM_10       : ------------------------------------------------------------------------------------------EPTTYIQRLELTLFCFTFLEIFLSGSTAI : 187
HS_10       : ------------------------------------------------------------------------------------------NSTVHIQRLELALLCFTVLELFLPVPTAV : 190
BT_10       : ------------------------------------------------------------------------------------------NDTVHIQRLVLALLCVTCVEVFLPGLMAI : 194
OA_10       : ------------------------------------------------------------------------------------------FYILHIQQLEIVLMVFSALEFLPAIAMVI : 196
HS_18       : -------------------------------------------------------------------------------------------VTTYSKAVSGGLLPFALLEFILTCVVSH : 265
BT_18       : --------------------------------------------------------------------------------------------VIDVKTVSGGLLPFVLLEFCLTCVVSH : 270
MM_18       : -------------------------------------------------------------------------------------------LMSSMKAAAGSILPFALLEFILTCIASH : 278
MD_8B       : ----------------------------------------------------------------------TSYYYY-----------YYYSGVASAMGISAVLFLFSGLELVVSIFSSH : 230
HS_8B       : -----------------------------------------------------------------------DYYPY-------------AWGVNPGMAISGVLLVFCLLEFGIACASSH : 204
BT_8B       : -----------------------------------------------------------------------LYDPP-----------SHTWGRVPGMAVSGLLLIFCILEVFIASISAH : 201
MM_8A       : -----------------------------------------------------------------------SYYPY-----------QENLGVRTGVAISSVLLIFCLLELSIASVSSH : 222
OA_8A       : -----------------------------------------------------------------------RYFTE-----------QGHLNHRSGAVLSTGLMLFSLLEFSIACVASH : 195
MM_13       : ----------------------------------------------------------------------VSY--------------RNYGQAKLGREVSRVLLCSYPLEFAIALLYSI : 149
BT_13       : ----------------------------------------------------------------------VSY--------------RNYGQAKLGRQISRVLLSFYPLEVSMALTYSI : 128
HS_13       : ----------------------------------------------------------------------VSY--------------RNYGQAKLGREVSRILLFFYGLEFSIALTHSI : 128
HS_12       : ------------------------------------------------------------------------------------------WAVLSGKGISATLMIFSLLEFFVACATAH : 222
MM_12       : ------------------------------------------------------------------------------------------WMVLSGRGIAGVLAIFSLLEFSIAGAMAY : 217
TG_MS4A15   : ----------------------------------------------------------------------------------------------SSFNYYSYLVLLAAPEV-------- : 139
AC_MS4Ax1   : ---------------------------------------------------------------------------------------ECYLGRTILTGSSAVLLLFSFLEFGITISTAH : 205
AC_MS4Ax2   : ---------------------------------------------------------------------------------------DMIMIQYVTTRIYFLLLVFNLLEFCITVSAAH : 187
DR_17A.15   : ----------------------------------------------------------------------YSYN-----------------HWNIFLRISGVLLVFAILEFFISISLSV : 189
DR_17A.3    : ----------------------------------------------------------------------YCND-----------------YWSLFLGVIGVLLVFAILEVFISISLSA : 186
DR_17A.2    : ----------------------------------------------------------------------YCYN-----------------YTTLLSGINGVLLAFAILEFFISICLSV : 188
DR_17A.5    : ------------------------------------------------------------------------YS-----------------YTSLLSGISCVLLVFAILEFCISICLFA : 175
DR_17A.1    : ----------------------------------------------------------------------YCYN-----------------YRSLFSGISCVLLLFAFLEFSISICLSA : 182
DR_17A.12   : -----------------------------------------------------------------------SLP-----------------YSCLFSGIRGVLLAFAVLELLISIFLSA : 184
DR_17A.4    : ----------------------------------------------------------------------YYFS-----------------ERTLLSGISGVLLVFAILEF-ISISLSV : 182
DR_17A.10   : ----------------------------------------------------------------------YCYI-----------------HLTLLSGISGVLLVFAILEFCISISLSV : 183
DR_17A.6    : ----------------------------------------------------------------------YCYD-----------------NWSLLSGISGVLLVFAILELCISICLSV : 180
DR_17A.11   : ----------------------------------------------------------------------YCYN-----------------YKTLFLGIRGVLLVFAILEFFISLSLSA : 193
DR_17A.7    : -----------------------------------------------------------------------CYN-----------------DVTIFIGISGVLLVFAILEFCISISLSV : 159
DR_17A.8    : -----------------------------------------------------------------------CYN-----------------DVTIVLGIKAVFLLFSILEFCISIYLSA : 186
DR_17A.9    : -----------------------------------------------------------------------CYE-----------------DTTIVLGINGIFLLFSILEFCISISLSA : 180
DR_17A.13   : ----------------------------------------------------------------------YCED-----------------YFNLLSGF---LLLFSILELFISIWLSA : 177
DR_17A.16   : ----------------------------------------------------------------------YCGEIE-----------R--MYMTLFSGISGVLLLFTILQFFISVYLCG : 195
DR_17A.17   : ----------------------------------------------------------------------YCAEME-----------L--KYTTLFTGINVVLLLFTILQFFISISLSA : 195
DR_17A.14   : ----------------------------------------------------------------------SSTDNI-----------ERQYRKALFAGISVVLLLFAILELVISICLSG : 199
DR_17B.1    : ----------------------------------------------------------------------YDYQ-------------FRYLYRTLSMGISGVLLVFAILECIISIFLSA : 194
DR_17B.2    : ----------------------------------------------------------------------YDY--------------YRYFYLTLSMGISGVLLVFAILECIISIFLSA : 193
DR_17C.2    : ----------------------------------------------------------------------GCGN-----------------LMKNKLGILGNLLVFSILQFIISICISG : 260
DR_17C.1    : ----------------------------------------------------------------------GNVG-----------------ICEFRQGIFGILLIFSILQFIISICISG : 233
OM_17C      : ----------------------------------------------------------------------LCYQ-----------------YWAQHQGISGIMVLFSLLEMMVTISVSA : 185
TN_MS4Ax3   : --------------------------------------------------------------LIEQKCKQHCFRKCTGL--------IEQKCKLLIDGILGTLVIFLVLELLICITAML : 191
TN_MS4Ax2   : --------------------------------------------------------------------FTLHYET------------TCRNIQNNQSHLFVEGMVIQVTLIAIAVTLAA : 170
SA_MS4Ax1   : ------------------------------------------------------------------------THCD-----------QLRPLYTATYYTFVVLLIFTILEFTVTIVVSA : 177
AT_MS4Ax1   : -------------------------------------------------------------------YNYYCVQP---------------AFLGMQTGVLAVLLILSLLEMGIAIATSA : 184
XT_MS4Ax2   : ----------------------------------------------------------------------------------------SFYPQTVITGMVAVLFILTIVELCVSLSTSI : 193
BT_1        : -----------------------------------------------------------------NPDENNSQSVE-----------YCARIRFVFLSNFAVMMIFAFLQKLVTAGTVE : 213
HS_1        : -----------------------------------------------------------------NPSEKNSPSTQ-----------YCYSIQSLFLGILSVMLIFAFFQELVIAGIVE : 213
MM_1        : -----------------------------------------------------------------NSSEKNSPSTQ-----------YCNSIQSVFLGILSAMLISAFFQKLVTAGIVE : 207
MD_1        : -----------------------------------------------------------------EPSKQSSPFHQ-----------YCLCLKSLYLGILAVMVIFTFLQNFLVACITE : 215
MD_15       : ----------------------------------------------------------------------------------------------VGRGYLAVLTIFTILEFFIAVIATY : 192
OA_15       : ----------------------------------------------------------------------------------------------VGRGYLAVLVIFTILEFFIAVVASH : 192
HS_15       : ----------------------------------------------------------------------------------------------VDRGYLAVLTIFTVLEFFTAVIAMH : 192
BT_15       : ----------------------------------------------------------------------------------------------VGRGYLAVLTIFTILEFFIAVIATH : 192
MM_15       : ----------------------------------------------------------------------------------------------VGRGYLAVLTIFTILEFFIAVIATH : 221
MM_5        : -----------------------------------------------------------------------GM--------------QCGVITILFIGILVMLMIFSIVELFISLFSSV : 186
HS_5        : -----------------------------------------------------------------------NS--------------QCKAVTVLFLGILITLMTFSIIELFISLPFSI : 186
BT_5        : -----------------------------------------------------------------------VS--------------ECHAVTTLFIGILIMLMAFSIIEFLISLSFSV : 186
HS_14       : QPG--------NKGREFVPDEQKQSILPSPKFSEEEIEP--LPPTLEKKPSENMSIQLDSTFKQMKDEDLQSAIVQ-----------PSQMQTKLLQDQAASLQVFPSHSALKLEDISP : 317
BT_14       : QHAGRRGSNSFNTSSLFMEDERQKYVPQTLSLYGEEVEVKHSPPILEQKSSE---IMMNT--EPLKDEDLQAAITQ-----------SPEEKPPLLQDQASKLQLFPSYSVKSVHDLPP : 355
MM_2        : ----------------------------------------------------------------------NCKNVTE---------DDGCFVASFTTELVLMMLFLTILAFCSAVLFTI : 192
HS_2        : -------------------------------------------------------------------SCQKFFETK-------------CFMASFSTEIVVMMLFLTILGLGSAVSLTI : 201
BT_2        : ----------------------------------------------------------------------SCEEVFVN---------DYCSLACFSTEVVAMILFLTILGFGSAVSLIA : 200
HS_6A       : -----------------------------------------------------------------SYFYHDSLYTT-----------DCYTAKASLAGTLSLMLICTLLEFCLAVLTAV : 205
HS_6E       : -----------------------------------------------------------------SYDYH-SPYTM-----------DCHRAKASLAGTLSLMLVSTVLEFCLAVLTAV : 140
MM_6D       : ------------------------------------------------------------------AYHFFSPEPLN----------SCFVAKAALTGVFSLMLISSVLELGLAVLTAT : 204
MM_11       : ---------------------------------------------------------------------------------------------------QQWKLAFTQLDT-TQD---- : 161
MM_6B       : -----------------------------------------------------------------YYQ---FLDRN-----------ECFAAKSVLAGVFSLMLISTMLELGLAVLTAM : 201
MM_6C       : -----------------------------------------------------------------YYQPFYNSDRS-----------ECAVTKSILTGALSVMLIISVLELGLALLSAM : 204
HS_7        : ------------------------------------------------------------------EYYYPIYEIK-----------DCLLTSVSLTGVLVVMLIFTVLELLLAAYSSV : 204
MM_7        : ------------------------------------------------------------------KSHHWKNEDK-----------NCYLAYVGAMSALGMMLLFTVLEVFLAGYSSI : 201
BT_7        : ------------------------------------------------------------------TYYYSIYDVK-----------DCLLTSVDLTGVLLVMLNFTVLELLAAAYASV : 205
HS_3        : -----------------------------------------------------------------------SPD-------------LCNYMGSISNGMVSLLLILTLLELCVTISTIA : 193
MM_3        : -----------------------------------------------------------------------SPD-------------VCISLGSSSDGLVSLMLILTLLELSVTISISA : 169
MM_4B       : ---------------------------------------------------------------------------------------------TITKGLDVLMLIFNMLEFCLAVSVSA : 168
MM_4C       : ---------------------------------------------------------------------------------------------TITKGLDILMLILNMLEFCIAVSISA : 168
MM_9        : ---------------------------------------------------------------------------------------------TITKGTDILMVMINMLEICNAEPSLL : 168
MM_4D       : ---------------------------------------------------------------------------------------------AIAS-LDVLMTILNMLEFCIAVSVSA : 167
HS_4E       : --------------------------------------------------------------------------SS-----------NCYMTMSILMGTDGMVLLLSVLEFCIAVSLSA : 208
HS_4A       : --------------------------------------------------------------------------SN-----------NCHGTMSILMGLDGMVLLLSVLEFCIAVSLSA : 170
MM_4x       : --------------------------------------------------------------------------SE-----------GCHMTLSILMGLDIVVVVLSVLEFCIGVSLSA : 185
AC_MS4Ax3   : ------------------------------------------------------------------------------------------SHVSIHLAFVALFLILTSLEFCITVIVTH : 201
BT_TM176B   : --------------------------------------------------------------------Y--QRRSSYSSWEEENCRRYMQMLMDLFLGIRALLLAICALQVTVSLASLG : 220
HS_TM176B   : --------------------------------------------------------------------YRWMRRSQENQWQKEECRAYMQMLRKLFTAIRALFLAVCVLKVIVSLVSLG : 226
MM_TM176B   : --------------------------------------------------------------------YGTVRYSDDSDWKTERCREYLNMMMNLFLAFCIMLTVVCILEIVVSVASLG : 221
OA_TM176B   : --------------------------------------------------------------------YSWQRQSG-SNWRQENCRKNMVLLVKLFGAVRGMMLAVFALLLAGSLASLG : 233
TN_TML1.3B  : ----------------------------------------------------------------------YSTETP--------------WQKLFAQKCWEKTQSTEGLLKCIYSLLII : 105
HS_TM176A   : ----------------------------------------------------------------------TPAPTQ------------SPEEVRRLHLCTSFMDMLKALFRTLQAMLLG : 197
BT_TM176A   : ---------------------------------------------------------------------TGAPTLSP--------------DLERLQQCTAYVNMLKALFISINAMLLG : 203
MM_TM176A   : -------------------------------------------------------------------------------------MPRTTPVPEEADRIALCIYYTSMLKTLLMSLQAM : 203
DR_TMLx     : -------------------------------------------------------------------YESRTPTPE-----------ESQRQVICEYYNNLNKMICGGLDIMMIVLSVL : 247
DR_TML1.2   : --------------------------------------------------------------------DSSTPSPE-----------QRSDRETCMKYMETNKMITGGLDIMIMVLSVL : 193
DR_TML1.3A  : --------------------------------------------------------------------YYSPNNGF-----------RGNSFDNCLYYRNLSKIILGGLDIMMIMLSVL : 301
TN_TML1.3A  : ----------------------------------------------------------------------YSTESP--------------WQKLFEEKCREKMLLTEWLLRCIIILLII : 191
DR_TML1.3B  : ----------------------------------------------------------------------DTTNN----------------NWITRALLDIAMIIFLVLQFCVTLSFSV : 163
BT_12       : ---------------------------------------------------------------------------------------------QLAGGMLLQYLLFSSVTELIVVSILL : 147
TN_MS4Ax1   : -------------------------------------------------------------------------------------------TREQFVWVQSLLLASSGCMLVILICLCT : 110
XT_MS4Ax1   : ----------------------------------------------------------------------HVIH-------------ARFILGKLVQGANIAMLIASVSSLCVALCIAY : 138
TN_MS4Ax4   : ---------------------------------------------------------------------FYSYHEQ-----------MNYDCPTEVLEAYTWSVIFLLLLYNIGAIFLH : 161
SP_out      : ----------------------------------------------------------------------YSIDSI-----------CEYNRSVGAIFVHTILAILGLVEFINCIVSAA : 262
```

```
                600        *        620        *        640        *        660        *        680        *        700        *
MM_10      : ---TAYRMKRLQAEDKDDTPFIPDT----PMELKGL-----SLGPPPSYKDVAQ---------------------------------------------------------GHSSSDTG : 237
HS_10      : ---TAWRGDCPSAKN-DDACLVPNT----PLHLKGL-----PVEPPPSYQSVIQ---------------------------------------------------------GDAQHKQH : 239
BT_10      : ---VAYRDARLSAEE-DDLPLVPDS----PLELKEQ------------------------------------------------------------------------------SMMPP : 227
OA_10      : ---FSCTAKSHSSDELETSFVDAGE----IPAEVTM-----RHVDPPPS-----------------------------------------------------------------YEEVV : 238
HS_18      : ---FGCQATCCRQFENVAVIP---------TVFSFN-----PANTTTSPVNATTGPVNAA-------------------------------------------TGPVSATNGPVNTTIH : 324
BT_18      : ---FGCQATCWNQFVNRTEVP---------TIVIAN-----PANTPT----------------------------------------------------------------GPFNATYS : 308
MM_18      : ---FGCQAVCCAHFQNMTMFP---------TIFGGN-----TVNTTT----------------------------------------------------------------GPGNTTIP : 316
MD_8B      : ---AGCQV-VSSQTNQVAVAVAIPV----PGTHGAP-----PMGIPQ-------------------------------------------------------------------PAVNS : 269
HS_8B      : ---FGCQL-VCCQSSNVSVIY--------PNIYAAN-----PVITPE-------------------------------------------------------------------PVTSP : 239
BT_8B      : ---FGCQL-TCYQTNNVGVVI--------PNVYVTN-----PAVIPE-------------------------------------------------------------------PENSP : 236
MM_8A      : ---FGCQV-ACCHYNNPGVVI--------PNVYAAN-----PVVIPE-------------------------------------------------------------------PPNPI : 257
OA_8A      : ---YGCLVGSCCGGGQVTMII--------PNDPTAT-----PMMAPD-------------------------------------------------------------------PMPSA : 231
MM_13      : ---SSCAYLPLSSIVKSLVRKTWRL----SSLAAWR-----QMIWLEA----------------------------------------------------------------GNQEETL : 192
BT_13      : ---FGCV---------GLCRK---------------------------------------------------------------------------------------------KEDAR : 142
HS_13      : ---YSCS---------NLFRR----------------------------------------------------------------------------------------------QNDL : 141
HS_12      : ---FANQANTT-TNMSVLVIP---------NMYESN-----PVTPAS-------------------------------------------------------------------SSAPP : 256
MM_12      : ---FAHQGILR-CNRSVPVAP---------AVYVAN-----PLMRES-------------------------------------------------------------------PSAPP : 251
TG_MS4A15  : ----------------------------------------------------------------------------------------------------------------------- :   -
AC_MS4Ax1  : ---FGCQAACCPSNQQPMVLYPTLS-------------------------------------------------------------------------------------------VEV : 230
AC_MS4Ax2  : ---FGCRATCCKSDPESVAYVPCTVS---GDAAMIP----------------------------------------------------------------------------EIYPSAP : 224
DR_17A.15  : ---FACKVACCCKPK-------------------------------------------------------------------------------------------------------- : 201
DR_17A.3   : ---FACKATSCCKPKIPVDNNPLIQ----HPPAEIP-----QQYIQCPQEVP------------------------------------------------------------EPCFQHP : 233
DR_17A.2   : ---FACKVTCCCKPKIHVSSNPLIQ----RPPAETP-----QQYSQCPQEVP------------------------------------------------------------EPCFQHP : 235
DR_17A.5   : ---FACKVTCCCKPKIPVSSNPLIQ----HPPAEMP-----PQYIQCPQEVP------------------------------------------------------------EPCFQHP : 222
DR_17A.1   : ---FACKVTCCYYPQIPVSSNPLIQ----HPPAEMP-----QQYIQCPQEVP------------------------------------------------------------EPCFQHP : 229
DR_17A.12  : ---FGCKVTCCYYPQIPVDSNLLIQ---------------------CPQEIP------------------------------------------------------------EPCFQHP : 219
DR_17A.4   : ---FACKATSCCKPKIPVNSNPLIQ----HPPAEMP-----PQYIQCPQEVP------------------------------------------------------------EPCFQHP : 229
DR_17A.10  : ---FACKVTCCYYPQISVVNNPLIQ----HPPAEIP-----QQYIQCPQEVP------------------------------------------------------------EPCFQHP : 230
DR_17A.6   : ---FACKVTCCYYPQIPVVNNPLIQ----RPPAENP-----QQYIQCPQEIP------------------------------------------------------------EPYFQHP : 227
DR_17A.11  : ---FGCKVTCCYYPQISVVNNPLIQ----HPPAENP-----QQYIQYPQEVP------------------------------------------------------------EPCFQHP : 240
DR_17A.7   : ---FACKVACCCKPKIPVENNILIQ----HTPAEIP-----QQYIQCPQEVP------------------------------------------------------------EPYFQHP : 206
DR_17A.8   : ---FACKVTSCCKPQIPEYRQPLIQ----HPPAEIP-----QQYLQCPQGIP------------------------------------------------------------QEYFQHP : 233
DR_17A.9   : ---FACKVACCCKPKIPEDRDPLIQ----HPPAEIP-----QQYIQYPQGIP------------------------------------------------------------QEYFQCP : 227
DR_17A.13  : ---FGCKVTCCNFP--------------------------------------------------------------------------------------------------------- : 188
DR_17A.16  : ---FASKFTSCCHP--QVPSISPVA----SPQPNYF-----Q--NTSEIPVAS-----------------------------------------------------------NPFFQHP : 239
DR_17A.17  : ---FACKVSCCCNPEVQVPTVSQVV----SPRPHHV-----HGLNTSGIAVVS------------------------------------------------------NHYVQQPPAESL : 248
DR_17A.14  : ---FACNADSCCNTQAPTVS-----------QAVYP-----QITYTSEVPVAS-----------------------------------------------------------NPLIQHP : 240
DR_17B.1   : ---SACKAT-CCSPQIAVISNPATH---------------------------------------------------------------------------------------------- : 215
DR_17B.2   : ---SACKAT-CCSPQIAVISNPSIH-----------------------------------------------------------------------------------------PAESA : 219
DR_17C.2   : ---FACKVTCNIDSAVVNVALNQAS----RDCDLRR-----DDGNHRGLKEVV-----------------------------------------------------------DHNPRLG : 308
DR_17C.1   : ---FACKATCNSES-VVNVALNQSI---------------------------------------------------------------------------------------------- : 254
OM_17C     : ---FACKAVCHSPPEQVFIVGSHLP----QGGPVPS-----QTTFDIPPNTYE-----------------------------------------------------------TLRTPKG : 233
TN_MS4Ax3  : ---FGLSALAAGGNQASSRSPAYPQ----TRPPPPP-----AAPRPAQVEVVVT----------------------------------------------------------EAVSEVL : 240
TN_MS4Ax2  : ---YCCKVANCCAPAPKMPVITVQA---------------------------------------------------------------------------------------------G : 193
SA_MS4Ax1  : ---FVCKSTRCCHPYSSMPTILLDT----SMTEEE------------------------------------------------------------------------------------ : 205
AT_MS4Ax1  : ---FGCKSVCHAGSQASQPMITVQY-----------------------------------------------------------------------------------------ANSQD : 211
XT_MS4Ax2  : ---FACKGLCRTSFNEMTVVVYQTAT-------------------------------------------------------------------------------------MTTDHS : 222
BT_1       : ---NEWKKLCSRPKANVVLLSAEEK----KEQVIEI-----KEEVVEQIEVAEQIEISSL-------------------------------------------PKNEEDIEIIPVQEEE : 277
HS_1       : ---NEWKRTCSRPKSNIVLLSAEEK----KEQTIEI-----KEEVVGLTETSSQ-------------------------------------------------PKNEEDIEIIPIQEEE : 271
MM_1       : ---NEWKRMCTRSKSNVVLLSAGEK----NEQTIKM-----KEEIIELSGVSSQ-------------------------------------------------PKNEEEIEIIPVQEEE : 265
MD_1       : ---NEWQTLCSGAQTSVILLSTEERKEQLVQLTVVPSQ---PKEEDDLETLPAQEEEKE------------------------------------------------------PNFPDP : 274
MD_15      : ---FGCQATRSNPNRSMVFLP---------NAFSTD-----FNIPSP-------------------------------------------------------------------AATPP : 227
OA_15      : ---FGCQAARSGSNAPVIFLP---------NAFSSD-----FGIPGP-------------------------------------------------------------------AATPP : 227
HS_15      : ---FGCQAIHAQASAPVIFLP---------NAFSAD-----FNIPSP-------------------------------------------------------------------AAPAP : 227
BT_15      : ---FGCQATRAQAHAPVIFLP---------NAFSTD-----FNIPSP-------------------------------------------------------------------AASPP : 227
MM_15      : ---FGCQATRAQTNASVIFLP---------NAFGTD-----FNIPSP-------------------------------------------------------------------AVSPP : 256
MM_5       : ---LGCYTEG---RGCC------------------------------------------------------------------------------------------------------ : 197
HS_5       : ---LGCHSEDCDCEQCC------------------------------------------------------------------------------------------------------ : 200
BT_5       : ---MRGPTECGDCEEWDGAEL-------------------------------------------------------------------------------------------------- : 204
HS_14      : -EDLPSQALPVEGLSEQTMPSKS------TSSHVKQ-----SSNLTANDLPPQGILSQDTSSQDMLFHDMTSQDMQSLDMLSQDTPSHAMPPQDIPSQDMLSQALSAHAILPEASTSHI : 424
BT_14      : -QDLPYQAPRAKTSPEQDLLSEA------STSHVTE-----SHGITSQDKESQGIPLQNT---------------QSHDMLSDYLPSPDMPAQDLAFQ---RQALPLQAIPFGATSLLS : 444
MM_2       : Y-RIGQELESKKVP--DDRLYEELN-------------------------------------------------------------------------------------------VYS : 217
HS_2       : C-GAGEELKGNKVP--EDRVYEELN-------------------------------------------------------------------------------------------IYS : 226
BT_2       : Y-GIGEILEGNQIP--EDRLYEEVN-------------------------------------------------------------------------------------------IYA : 225
HS_6A      : ---LRWKQAYSDFPGSVLFLPHSYI----GNSGMSS------------------------------------------------------------------------------KMTHD : 239
HS_6E      : ---LQWKQTV------------------------------------------------------------------------------------------------------------- : 147
MM_6D      : ---LWWKQSSSAFSGNVIFLSQNSK----NKSSVSS----------------------------------------------------------------------------------- : 233
MM_11      : ----AYQFFSPEPLN---------------SCFVG------------------------------------------------------------------------------QSWSDW : 183
MM_6B      : ---LWWKQSHSNIPGNVMFLPHSSN----NDSNMES----------------------------------------------------------------------------------- : 230
MM_6C      : ---LWLREG-------------------------------------------------------------------------------------------------------------- : 210
HS_7       : ---FWWKQLYSNNPGSSFSSTQSQD---------------------------------------------------------------------------------------------- : 226
MM_7       : ---FWWKQVYSNKPGGTFFLPQSQD---------------------------------------------------------------------------------------------H : 224
BT_7       : ---FWWRR-------STVTALRSSS----HE---------------------------------------------------------------------------------------- : 222
HS_3       : ---MWCNANCCNSREE-ISSPPNSV---------------------------------------------------------------------------------------------- : 214
MM_3       : ---MWCLGNVCGLREA-ITSPPNSV----ESG-----------ILPEGSD-------------------------------------------------------------------SE : 202
MM_4B      : ---FGCEASCCNSREVLVVLPSNPV----ETVMAPP-----MTLQPLL-PSEHQ------------------------------------------------------------GTNVP : 214
MM_4C      : ---FGCKASCCNSSEVLVVLPSNPA----VTVMAPP-----VTLQPLP-PSEHQ------------------------------------------------------------GKNVP : 214
MM_9       : ---LDVKLAGVLQR-GLVVLPSNPA----VTGGGPPPGWTASTHWPPH-QETPK------------------------------------------------------------GKKCS : 218
MM_4D      : ---FGCKASCCNSSEVLVVLPSNSA----VTVTAPP-----MILQPLP-PSECQ------------------------------------------------------------GKNVP : 213
HS_4E      : ---FGCKVLCCSPSEVVLITPSNSH----MAEIASP----------------------------------------------------------------------------------T : 238
HS_4A      : ---FGCKVLCCTPGGVVLILPSHSH----MAETASP---------------------------------------------------------------------------------TP : 201
MM_4x      : ---FGCRVMCCNPGGVMIIMPSNPT----KAETANP-------------------------------------------------------------------------------VTLQ : 218
AC_MS4Ax3  : ---FGCRANCCTDDTAIVYVPCTIN----------------------------------------------------------------------------------------GENTIP : 229
BT_TM176B  : ---VGLQSFCGQSS-RALQDEEGSE----RNLLG--------------------------------------------------------------------------------ENSVP : 251
HS_TM176B  : ---VGLRNLCGQSS-QPLN-EEGSE----KRLLG--------------------------------------------------------------------------------ENSVP : 256
MM_TM176B  : ---LSLRSMYGRSS-QALN-EEESE----RKLLD--------------------------------------------------------------------------------GHPAP : 251
OA_TM176B  : ---LGLRCLCCRADNLELAGACPLR----GDVWLSA-----PSAPGVTLWSINDTDKHMH-------------------------------------------SQPQHWTEFRVIASNS : 297
TN_TML1.3B : ---LSFMEVFVSISSVVLGIKAFGS----SPGR-------------------------------------------------------------------------------ENRSNED : 138
HS_TM176A  : ---VWILLLLASLTPLWLYCWRMFP----TKG------------------------------------------------------------------------------------KRD : 225
BT_TM176A  : ---VWVLLLLASLLPLCLCCWRRYR----RKE------------------------------------------------------------------------------------KRD : 231
MM_TM176A  : ---LLGIWVLLLLASLTPVCVYIWK----RFFTKA------------------------------------------------------------------------------------ : 231
DR_TMLx    : ---QLCVAISLCVLTLKTLCKKTE----------------------------------------------------------------------------------------NAK--ED : 273
DR_TML1.2  : ---QLCVTISFCVLTGKVLCTKTE----------------------------------------------------------------------------------------DAEMVKD : 221
DR_TML1.3A : ---QLCVTISFCVLTGKVLCNKSE----------------------------------------------------------------------------------------DGEILED : 329
TN_TML1.3A : ---LSVMELCVSVSSAVLGIKALRN----SSAR-------------------------------------------------------------------------------ENKSNED : 224
DR_TML1.3B : ---LTLKEICEKN------------------------------------------------------------------------------------------------------TVKH : 177
BT_12      : C--WIVRALKQSEPKKESYTLSESS----VSS--------------------------------------------------------------------------------------- : 173
TN_MS4Ax1  : ---FARLALKSTRNQVIVHHVRPSQ---------------------------------------------------------------------------------------------- : 132
XT_MS4Ax1  : ---MGCRSLPQCMCYDDITGMDWLH----PEDEQPQ-----TVELVCTFRGGD-----------------------------------------------------------ERIFNSA : 186
TN_MS4Ax4  : ----GFLSLSAIKTLKAT----------------------------------------------------------------------------------------------------- : 175
SP_out     : ---YCCGGHSCCCAKAPTNQGTAST----ATTVQYG-----QAQP---------------------------------------------------------------------MTVAA : 300
```

```
                720         *        740         *        760         *        780         *        800         *        820         *
MM_10      : RALATSSG-----------LLLASDSFHQALLHTGPRTLRK------------------------------------------------------------------------------ : 267
HS_10      : QRLREVKQ--------------VAPDTWIVTDGAAIWAQTAN----------------------------------------------------------------------------- : 267
BT_10      : PSYEDVTQ--------------GDQQDEQERR--------------------------------------------------------------------------------------- : 245
OA_10      : GEEKRDVN--------------EGGLYP------------------------------------------------------------------------------------------- : 252
HS_18      : PVNTTTSP--------------VNTTTSPVNVTTGPVNANIGPVNVTTGPVNTTTAPAKATTSCVNAIHTSNVPPNPRTKK-------------------------------------- : 391
BT_18      : -TTGHVNV--------------ITSSANPTSPTN--AAAMVPPVPS------------------------------------------------------------------------- : 337
MM_18      : NSTTSIPV--------------QTTLPSNVPLEQ--VYQDVAQK--------------------------------------------------------------------------- : 344
MD_8B      : LVYSSEVN--------------SQQ---------------------------------------------------------------------------------------------- : 280
HS_8B      : PSYSSEIQ--------------ANK---------------------------------------------------------------------------------------------- : 250
BT_8B      : PNYSDVPR--------------DK----------------------------------------------------------------------------------------------- : 246
MM_8A      : PSYSEVVQ--------------DSR---------------------------------------------------------------------------------------------- : 268
OA_8A      : PDFSNTVG--------------TTSSGHTLRA--------------------------------------------------------------------------------------- : 249
MM_13      : ESVTEVVE--------------GNS---------------------------------------------------------------------------------------------- : 203
BT_13      : TADTEEVE--------------DAF---------------------------------------------------------------------------------------------- : 153
HS_13      : TSVTEEAE--------------STP---------------------------------------------------------------------------------------------- : 152
HS_12      : RCNNYSAN--------------APK---------------------------------------------------------------------------------------------- : 267
MM_12      : IYDNIPDY--------------ATTQ--------------------------------------------------------------------------------------------- : 263
TG_MS4A15  : ----------------------------------------------------------------------------------------------------------------------- :   -
AC_MS4Ax1  : WWLLEKA---------------------------------------------------------------------------------------------------------------- : 237
AC_MS4Ax2  : PAYDVPELLLRG----------MKNRWKKL----------------------------------------------------------------------------------------- : 244
DR_17A.15  : ----------------------------------------------------------------------------------------------------------------------- :   -
DR_17A.3   : PAEAPPAY--------------TEGE--------------------------------------------------------------------------------------------- : 245
DR_17A.2   : PAETPPAY--------------TEGK--------------------------------------------------------------------------------------------- : 247
DR_17A.5   : PAESPPAY--------------TEGE--------------------------------------------------------------------------------------------- : 234
DR_17A.1   : PAEAPPAY--------------TEGK--------------------------------------------------------------------------------------------- : 241
DR_17A.12  : PAEAPPAY--------------TEANKCEST---------------------------------------------------------------------------------------- : 236
DR_17A.4   : PAESPPAY--------------TEANKCEST---------------------------------------------------------------------------------------- : 246
DR_17A.10  : PAEAPLAY--------------TEANKCESTSKYEAA--------------------------------------------------------------------------------- : 253
DR_17A.6   : PAEAPPAY--------------TEGKLM------------------------------------------------------------------------------------------- : 241
DR_17A.11  : PAEAPPAY--------------TEGK--------------------------------------------------------------------------------------------- : 252
DR_17A.7   : PAGFPPAY--------------TEGK--------------------------------------------------------------------------------------------- : 218
DR_17A.8   : PAEAPPAY--------------TEGKYM------------------------------------------------------------------------------------------- : 247
DR_17A.9   : PAEAPPAY--------------ASGKLM------------------------------------------------------------------------------------------- : 241
DR_17A.13  : ----------------------------------------------------------------------------------------------------------------------- :   -
DR_17A.16  : PAESPPEY--------------AECN--------------------------------------------------------------------------------------------- : 251
DR_17A.17  : PQYSEQPP--------------AESLLQYSQQPPAESLLQYSQQPAAETPPVYTERKLM------------------------------------------------------------ : 293
DR_17A.14  : PAGAPLQY--------------SQYK--------------------------------------------------------------------------------------------- : 252
DR_17B.1   : PAESAPIY--------------SNCE--------------------------------------------------------------------------------------------- : 227
DR_17B.2   : PIYCNCE---------------------------------------------------------------------------------------------------------------- : 226
DR_17C.2   : RILSTCCG--------------SHGGVQSMRP--------------------------------------------------------------------------------------- : 326
DR_17C.1   : ----------------------------------------------------------------------------------------------------------------------- :   -
OM_17C     : AEEGLPAY--------------DNNRPPQYSPAIIS----------------------------------------------------------------------------------- : 255
TN_MS4Ax3  : EEASTPGG--------------AQEPGISHA---------------------------------------------------------------------------------------- : 257
TN_MS4Ax2  : PAQT------------------------------------------------------------------------------------------------------------------- : 197
SA_MS4Ax1  : ----------------------------------------------------------------------------------------------------------------------- :   -
AT_MS4Ax1  : PRFHPTQN--------------AGILLG------------------------------------------------------------------------------------------- : 225
XT_MS4Ax2  : STAAGDMK--------------PSQDG-------------------------------------------------------------------------------------------- : 235
BT_1       : EEEAEMNF--------------PEPPQDQEDSPIENDSVP------------------------------------------------------------------------------- : 303
HS_1       : EEETETNF--------------PEPPQDQESSPIENDSSP------------------------------------------------------------------------------- : 297
MM_1       : EEEAEINF--------------PAPPQEQESLPVENEIAP------------------------------------------------------------------------------- : 291
MD_1       : PQ--ESSP--------------TEDACSS------------------------------------------------------------------------------------------ : 287
MD_15      : PAYDNVAY--------------TSKETSE------------------------------------------------------------------------------------------ : 242
OA_15      : PAYDNVAY--------------LPKELNE------------------------------------------------------------------------------------------ : 242
HS_15      : PAYDNVAY--------------AQGVV-------------------------------------------------------------------------------------------- : 240
BT_15      : PAYDNVAY--------------IPKESSE------------------------------------------------------------------------------------------ : 242
MM_15      : PAYDNVAY--------------MPKESSE------------------------------------------------------------------------------------------ : 271
MM_5       : ----------------------------------------------------------------------------------------------------------------------- :   -
HS_5       : ----------------------------------------------------------------------------------------------------------------------- :   -
BT_5       : ----------------------------------------------------------------------------------------------------------------------- :   -
HS_14      : VQFPEIQHLLQQPPDLQPENTEPQNQQILQMSYQDIRSEVMEETKEWKSEEELHRRKSSRRHSLNQQTKALQYLRRHSLDVQAKGQKSSKRHSLDQQSKGWQSPKQKSLDQQIK----- : 538
BT_14      : VQSSNMQHLDRRSLDFQLHNIQSQEQRAVHLLYQDIKSEVMLMTEEWKPEEELQSRRPSKQHSQLQQSKGCPCAKQKALDVYIQDQPHPRRKSLDKHIKSWLSPKKQFIDKEIQVSQTI : 563
MM_2       : PIYSELED--------------KG----ETSSPVD---------S-------------------------------------------------------------------------- : 235
HS_2       : ATYSELED--------------PG----EMSPPID---------L-------------------------------------------------------------------------- : 244
BT_2       : PIYSELEE--------------RE----EPTSPAD---------S-------------------------------------------------------------------------- : 243
HS_6A      : CGYEELLT--------------S------------------------------------------------------------------------------------------------ : 248
HS_6E      : ----------------------------------------------------------------------------------------------------------------------- :   -
MM_6D      : ESLCNPTY--------------ENILTS------------------------------------------------------------------------------------------- : 247
MM_11      : S-----LF--------------TDAK--------------------------------------------------------------------------------------------- : 190
MM_6B      : KVLCNPSY--------------EEQLVC------------------------------------------------------------------------------------------- : 244
MM_6C      : -VLTSLRM--------------------------------------------------------------------------------------------------------------- : 217
HS_7       : -HIQQVKK--------------SSSRSWI------------------------------------------------------------------------------------------ : 240
MM_7       : TQLVKS----------------NLLQ--------------------------------------------------------------------------------------------- : 234
BT_7       : ----------------------------------------------------------------------------------------------------------------------- :   -
HS_3       : ----------------------------------------------------------------------------------------------------------------------- :   -
MM_3       : NLNTQPQA--------------SEE---------------------------------------------------------------------------------------------- : 213
MM_4B      : -GNVYKNH--------------PGEIV-------------------------------------------------------------------------------------------- : 226
MM_4C      : -ENVYKNH--------------SEEIV-------------------------------------------------------------------------------------------- : 226
MM_9       : RRNVYKET--------------TQKK--------------------------------------------------------------------------------------------- : 230
MM_4D      : -ENLYRNQ--------------PGEIV-------------------------------------------------------------------------------------------- : 225
HS_4E      : PLKTV------------------------------------------------------------------------------------------------------------------ : 243
HS_4A      : LNEV------------------------------------------------------------------------------------------------------------------- : 205
MM_4x      : SGLMPPEH--------------Q-ERNVP-ENMH------------------------------------------------------------------------------------- : 236
AC_MS4Ax3  : TEKMWLPS--------------------------------------------------------------------------------------------------------------- : 237
BT_TM176B  : PSPSKEKT--------------TAVIVL------------------------------------------------------------------------------------------- : 265
HS_TM176B  : PSPSREQT--------------STAIVL------------------------------------------------------------------------------------------- : 270
MM_TM176B  : ASPAKEKI--------------PAIL--------------------------------------------------------------------------------------------- : 263
OA_TM176B  : LEDKSNAL--------------SVSASLKVSLLGGWSSSSS------------------------------------------------------------------------------ : 324
TN_TML1.3B : AERCTPLL--------------AEASGDPTP---------------------------------------------------------------------------------------- : 155
HS_TM176A  : QKEMLEVS--------------GI----------------------------------------------------------------------------------------------- : 235
BT_TM176A  : LPLEETVR--------------SE----------------------------------------------------------------------------------------------- : 241
MM_TM176A  : ETEEKKLL--------------GAAVI-------------------------------------------------------------------------------------------- : 244
DR_TMLx    : LEHGKPLL--------------DDDIAGAA----------------------------------------------------------------------------------------- : 289
DR_TML1.2  : PELNKPLL--------------EDAIAAN------------------------------------------------------------------------------------------ : 236
DR_TML1.3A : PELKKGLL--------------EDAIATIA----------------------------------------------------------------------------------------- : 345
TN_TML1.3A : PEQCTPLL--------------AEASGDPTP---------------------------------------------------------------------------------------- : 241
DR_TML1.3B : LEFYKPLK--------------EELTVSHLC---------------------------------------------------------------------------------------- : 194
BT_12      : ----------------------------------------------------------------------------------------------------------------------- :   -
TN_MS4Ax1  : --TTITSY--------------------------------------------------------------------------------------------------------------- : 138
XT_MS4Ax1  : SQFTDCNT--------------ETEEEFSRPPPYVRFS--------------------------------------------------------------------------------- : 210
TN_MS4Ax4  : ----------------------------------------------------------------------------------------------------------------------- :   -
SP_out     : NRYAPGPE--------------SGK---------------------------------------------------------------------------------------------- : 311
```

```
                    840         *       860         *       880         *       900         *       920         *       940         *
MM_10       : ----------------------------------------------------------------------------------------------------------------------- :   -
HS_10       : ----------------------------------------------------------------------------------------------------------------------- :   -
BT_10       : ----------------------------------------------------------------------------------------------------------------------- :   -
OA_10       : ----------------------------------------------------------------------------------------------------------------------- :   -
HS_18       : ----------------------------------------------------------------------------------------------------------------------- :   -
BT_18       : ----------------------------------------------------------------------------------------------------------------------- :   -
MM_18       : ----------------------------------------------------------------------------------------------------------------------- :   -
MD_8B       : ----------------------------------------------------------------------------------------------------------------------- :   -
HS_8B       : ----------------------------------------------------------------------------------------------------------------------- :   -
BT_8B       : ----------------------------------------------------------------------------------------------------------------------- :   -
MM_8A       : ----------------------------------------------------------------------------------------------------------------------- :   -
OA_8A       : ----------------------------------------------------------------------------------------------------------------------- :   -
MM_13       : ----------------------------------------------------------------------------------------------------------------------- :   -
BT_13       : ----------------------------------------------------------------------------------------------------------------------- :   -
HS_13       : ----------------------------------------------------------------------------------------------------------------------- :   -
HS_12       : ----------------------------------------------------------------------------------------------------------------------- :   -
MM_12       : ----------------------------------------------------------------------------------------------------------------------- :   -
TG_MS4A15   : ----------------------------------------------------------------------------------------------------------------------- :   -
AC_MS4Ax1   : ----------------------------------------------------------------------------------------------------------------------- :   -
AC_MS4Ax2   : ----------------------------------------------------------------------------------------------------------------------- :   -
DR_17A.15   : ----------------------------------------------------------------------------------------------------------------------- :   -
DR_17A.3    : ----------------------------------------------------------------------------------------------------------------------- :   -
DR_17A.2    : ----------------------------------------------------------------------------------------------------------------------- :   -
DR_17A.5    : ----------------------------------------------------------------------------------------------------------------------- :   -
DR_17A.1    : ----------------------------------------------------------------------------------------------------------------------- :   -
DR_17A.12   : ----------------------------------------------------------------------------------------------------------------------- :   -
DR_17A.4    : ----------------------------------------------------------------------------------------------------------------------- :   -
DR_17A.10   : ----------------------------------------------------------------------------------------------------------------------- :   -
DR_17A.6    : ----------------------------------------------------------------------------------------------------------------------- :   -
DR_17A.11   : ----------------------------------------------------------------------------------------------------------------------- :   -
DR_17A.7    : ----------------------------------------------------------------------------------------------------------------------- :   -
DR_17A.8    : ----------------------------------------------------------------------------------------------------------------------- :   -
DR_17A.9    : ----------------------------------------------------------------------------------------------------------------------- :   -
DR_17A.13   : ----------------------------------------------------------------------------------------------------------------------- :   -
DR_17A.16   : ----------------------------------------------------------------------------------------------------------------------- :   -
DR_17A.17   : ----------------------------------------------------------------------------------------------------------------------- :   -
DR_17A.14   : ----------------------------------------------------------------------------------------------------------------------- :   -
DR_17B.1    : ----------------------------------------------------------------------------------------------------------------------- :   -
DR_17B.2    : ----------------------------------------------------------------------------------------------------------------------- :   -
DR_17C.2    : ----------------------------------------------------------------------------------------------------------------------- :   -
DR_17C.1    : ----------------------------------------------------------------------------------------------------------------------- :   -
OM_17C      : ----------------------------------------------------------------------------------------------------------------------- :   -
TN_MS4Ax3   : ----------------------------------------------------------------------------------------------------------------------- :   -
TN_MS4Ax2   : ----------------------------------------------------------------------------------------------------------------------- :   -
SA_MS4Ax1   : ----------------------------------------------------------------------------------------------------------------------- :   -
AT_MS4Ax1   : ----------------------------------------------------------------------------------------------------------------------- :   -
XT_MS4Ax2   : ----------------------------------------------------------------------------------------------------------------------- :   -
BT_1        : ----------------------------------------------------------------------------------------------------------------------- :   -
HS_1        : ----------------------------------------------------------------------------------------------------------------------- :   -
MM_1        : ----------------------------------------------------------------------------------------------------------------------- :   -
MD_1        : ----------------------------------------------------------------------------------------------------------------------- :   -
MD_15       : ----------------------------------------------------------------------------------------------------------------------- :   -
OA_15       : ----------------------------------------------------------------------------------------------------------------------- :   -
HS_15       : ----------------------------------------------------------------------------------------------------------------------- :   -
BT_15       : ----------------------------------------------------------------------------------------------------------------------- :   -
MM_15       : ----------------------------------------------------------------------------------------------------------------------- :   -
MM_5        : ----------------------------------------------------------------------------------------------------------------------- :   -
HS_5        : ----------------------------------------------------------------------------------------------------------------------- :   -
BT_5        : ----------------------------------------------------------------------------------------------------------------------- :   -
HS_14       : --------------------------------DWLSPKR-----HSVDKQAQLNQTKEQLP------DQQAEDQQAKGEQYPEGQSKDGQVKDQQ-----------------TDKEQNS : 597
BT_14       : LQLPDQQAENLRIQEEKPPRQLYQDMQIQEYHDWQSPDRRVQTWQSLGQQSQEWRTQDWKSKEWEKRERQLEMQHSQNWDFQAWQTQDLQVKESRKQRSLFQETQTLHATIPADLDEQS : 682
MM_2        : ----------------------------------------------------------------------------------------------------------------------- :   -
HS_2        : ----------------------------------------------------------------------------------------------------------------------- :   -
BT_2        : ----------------------------------------------------------------------------------------------------------------------- :   -
HS_6A       : ----------------------------------------------------------------------------------------------------------------------- :   -
HS_6E       : ----------------------------------------------------------------------------------------------------------------------- :   -
MM_6D       : ----------------------------------------------------------------------------------------------------------------------- :   -
MM_11       : ----------------------------------------------------------------------------------------------------------------------- :   -
MM_6B       : ----------------------------------------------------------------------------------------------------------------------- :   -
MM_6C       : ----------------------------------------------------------------------------------------------------------------------- :   -
HS_7        : ----------------------------------------------------------------------------------------------------------------------- :   -
MM_7        : ----------------------------------------------------------------------------------------------------------------------- :   -
BT_7        : ----------------------------------------------------------------------------------------------------------------------- :   -
HS_3        : ----------------------------------------------------------------------------------------------------------------------- :   -
MM_3        : ----------------------------------------------------------------------------------------------------------------------- :   -
MM_4B       : ----------------------------------------------------------------------------------------------------------------------- :   -
MM_4C       : ----------------------------------------------------------------------------------------------------------------------- :   -
MM_9        : ----------------------------------------------------------------------------------------------------------------------- :   -
MM_4D       : ----------------------------------------------------------------------------------------------------------------------- :   -
HS_4E       : ----------------------------------------------------------------------------------------------------------------------- :   -
HS_4A       : ----------------------------------------------------------------------------------------------------------------------- :   -
MM_4x       : ----------------------------------------------------------------------------------------------------------------------- :   -
AC_MS4Ax3   : ----------------------------------------------------------------------------------------------------------------------- :   -
BT_TM176B   : ----------------------------------------------------------------------------------------------------------------------- :   -
HS_TM176B   : ----------------------------------------------------------------------------------------------------------------------- :   -
MM_TM176B   : ----------------------------------------------------------------------------------------------------------------------- :   -
OA_TM176B   : ----------------------------------------------------------------------------------------------------------------------- :   -
TN_TML1.3B  : ----------------------------------------------------------------------------------------------------------------------- :   -
HS_TM176A   : ----------------------------------------------------------------------------------------------------------------------- :   -
BT_TM176A   : ----------------------------------------------------------------------------------------------------------------------- :   -
MM_TM176A   : ----------------------------------------------------------------------------------------------------------------------- :   -
DR_TMLx     : ----------------------------------------------------------------------------------------------------------------------- :   -
DR_TML1.2   : ----------------------------------------------------------------------------------------------------------------------- :   -
DR_TML1.3A  : ----------------------------------------------------------------------------------------------------------------------- :   -
TN_TML1.3A  : ----------------------------------------------------------------------------------------------------------------------- :   -
DR_TML1.3B  : ----------------------------------------------------------------------------------------------------------------------- :   -
BT_12       : ----------------------------------------------------------------------------------------------------------------------- :   -
TN_MS4Ax1   : ----------------------------------------------------------------------------------------------------------------------- :   -
XT_MS4Ax1   : ----------------------------------------------------------------------------------------------------------------------- :   -
TN_MS4Ax4   : ----------------------------------------------------------------------------------------------------------------------- :   -
SP_out      : ----------------------------------------------------------------------------------------------------------------------- :   -
```

```
                    960         *       980         *      1000         *      1020         *      1040         *      1060         *
MM_10        : ----------------------------------------------------------------------------------------------------------------------- :   -
HS_10        : ----------------------------------------------------------------------------------------------------------------------- :   -
BT_10        : ----------------------------------------------------------------------------------------------------------------------- :   -
OA_10        : ----------------------------------------------------------------------------------------------------------------------- :   -
HS_18        : ----------------------------------------------------------------------------------------------------------------------- :   -
BT_18        : ----------------------------------------------------------------------------------------------------------------------- :   -
MM_18        : ----------------------------------------------------------------------------------------------------------------------- :   -
MD_8B        : ----------------------------------------------------------------------------------------------------------------------- :   -
HS_8B        : ----------------------------------------------------------------------------------------------------------------------- :   -
BT_8B        : ----------------------------------------------------------------------------------------------------------------------- :   -
MM_8A        : ----------------------------------------------------------------------------------------------------------------------- :   -
OA_8A        : ----------------------------------------------------------------------------------------------------------------------- :   -
MM_13        : ----------------------------------------------------------------------------------------------------------------------- :   -
BT_13        : ----------------------------------------------------------------------------------------------------------------------- :   -
HS_13        : ----------------------------------------------------------------------------------------------------------------------- :   -
HS_12        : ----------------------------------------------------------------------------------------------------------------------- :   -
MM_12        : ----------------------------------------------------------------------------------------------------------------------- :   -
TG_MS4A15    : ----------------------------------------------------------------------------------------------------------------------- :   -
AC_MS4Ax1    : ----------------------------------------------------------------------------------------------------------------------- :   -
AC_MS4Ax2    : ----------------------------------------------------------------------------------------------------------------------- :   -
DR_17A.15    : ----------------------------------------------------------------------------------------------------------------------- :   -
DR_17A.3     : ----------------------------------------------------------------------------------------------------------------------- :   -
DR_17A.2     : ----------------------------------------------------------------------------------------------------------------------- :   -
DR_17A.5     : ----------------------------------------------------------------------------------------------------------------------- :   -
DR_17A.1     : ----------------------------------------------------------------------------------------------------------------------- :   -
DR_17A.12    : ----------------------------------------------------------------------------------------------------------------------- :   -
DR_17A.4     : ----------------------------------------------------------------------------------------------------------------------- :   -
DR_17A.10    : ----------------------------------------------------------------------------------------------------------------------- :   -
DR_17A.6     : ----------------------------------------------------------------------------------------------------------------------- :   -
DR_17A.11    : ----------------------------------------------------------------------------------------------------------------------- :   -
DR_17A.7     : ----------------------------------------------------------------------------------------------------------------------- :   -
DR_17A.8     : ----------------------------------------------------------------------------------------------------------------------- :   -
DR_17A.9     : ----------------------------------------------------------------------------------------------------------------------- :   -
DR_17A.13    : ----------------------------------------------------------------------------------------------------------------------- :   -
DR_17A.16    : ----------------------------------------------------------------------------------------------------------------------- :   -
DR_17A.17    : ----------------------------------------------------------------------------------------------------------------------- :   -
DR_17A.14    : ----------------------------------------------------------------------------------------------------------------------- :   -
DR_17B.1     : ----------------------------------------------------------------------------------------------------------------------- :   -
DR_17B.2     : ----------------------------------------------------------------------------------------------------------------------- :   -
DR_17C.2     : ----------------------------------------------------------------------------------------------------------------------- :   -
DR_17C.1     : ----------------------------------------------------------------------------------------------------------------------- :   -
OM_17C       : ----------------------------------------------------------------------------------------------------------------------- :   -
TN_MS4Ax3    : ----------------------------------------------------------------------------------------------------------------------- :   -
TN_MS4Ax2    : ----------------------------------------------------------------------------------------------------------------------- :   -
SA_MS4Ax1    : ----------------------------------------------------------------------------------------------------------------------- :   -
AT_MS4Ax1    : ----------------------------------------------------------------------------------------------------------------------- :   -
XT_MS4Ax2    : ----------------------------------------------------------------------------------------------------------------------- :   -
BT_1         : ----------------------------------------------------------------------------------------------------------------------- :   -
HS_1         : ----------------------------------------------------------------------------------------------------------------------- :   -
MM_1         : ----------------------------------------------------------------------------------------------------------------------- :   -
MD_1         : ----------------------------------------------------------------------------------------------------------------------- :   -
MD_15        : ----------------------------------------------------------------------------------------------------------------------- :   -
OA_15        : ----------------------------------------------------------------------------------------------------------------------- :   -
HS_15        : ----------------------------------------------------------------------------------------------------------------------- :   -
BT_15        : ----------------------------------------------------------------------------------------------------------------------- :   -
MM_15        : ----------------------------------------------------------------------------------------------------------------------- :   -
MM_5         : ----------------------------------------------------------------------------------------------------------------------- :   -
HS_5         : ----------------------------------------------------------------------------------------------------------------------- :   -
BT_5         : ----------------------------------------------------------------------------------------------------------------------- :   -
HS_14        : KKQTQDQQTEDQPAQEKKSPKGQFQNVQAEGQQAQVEKVPKLLCQDS--------------------ESQIQQYQFWQFHKGNLQAGQPRTVNLLAKNPLTG----------------- : 679
BT_14        : QDVLQDSQHQNKDQQDLQSAGIQKEVMETDAVQTRDIKSEDMSCGKSQTPSDLQSEDAKSDFNCFSYQSSVQELQSTGSQKQDMETDAVQTRDIKSEDTSCGKSQSPTDLQLEDIKPDS : 801
MM_2         : ----------------------------------------------------------------------------------------------------------------------- :   -
HS_2         : ----------------------------------------------------------------------------------------------------------------------- :   -
BT_2         : ----------------------------------------------------------------------------------------------------------------------- :   -
HS_6A        : ----------------------------------------------------------------------------------------------------------------------- :   -
HS_6E        : ----------------------------------------------------------------------------------------------------------------------- :   -
MM_6D        : ----------------------------------------------------------------------------------------------------------------------- :   -
MM_11        : ----------------------------------------------------------------------------------------------------------------------- :   -
MM_6B        : ----------------------------------------------------------------------------------------------------------------------- :   -
MM_6C        : ----------------------------------------------------------------------------------------------------------------------- :   -
HS_7         : ----------------------------------------------------------------------------------------------------------------------- :   -
MM_7         : ----------------------------------------------------------------------------------------------------------------------- :   -
BT_7         : ----------------------------------------------------------------------------------------------------------------------- :   -
HS_3         : ----------------------------------------------------------------------------------------------------------------------- :   -
MM_3         : ----------------------------------------------------------------------------------------------------------------------- :   -
MM_4B        : ----------------------------------------------------------------------------------------------------------------------- :   -
MM_4C        : ----------------------------------------------------------------------------------------------------------------------- :   -
MM_9         : ----------------------------------------------------------------------------------------------------------------------- :   -
MM_4D        : ----------------------------------------------------------------------------------------------------------------------- :   -
HS_4E        : ----------------------------------------------------------------------------------------------------------------------- :   -
HS_4A        : ----------------------------------------------------------------------------------------------------------------------- :   -
MM_4x        : ----------------------------------------------------------------------------------------------------------------------- :   -
AC_MS4Ax3    : ----------------------------------------------------------------------------------------------------------------------- :   -
BT_TM176B    : ----------------------------------------------------------------------------------------------------------------------- :   -
HS_TM176B    : ----------------------------------------------------------------------------------------------------------------------- :   -
MM_TM176B    : ----------------------------------------------------------------------------------------------------------------------- :   -
OA_TM176B    : ----------------------------------------------------------------------------------------------------------------------- :   -
TN_TML1.3B   : ----------------------------------------------------------------------------------------------------------------------- :   -
HS_TM176A    : ----------------------------------------------------------------------------------------------------------------------- :   -
BT_TM176A    : ----------------------------------------------------------------------------------------------------------------------- :   -
MM_TM176A    : ----------------------------------------------------------------------------------------------------------------------- :   -
DR_TMLx      : ----------------------------------------------------------------------------------------------------------------------- :   -
DR_TML1.2    : ----------------------------------------------------------------------------------------------------------------------- :   -
DR_TML1.3A   : ----------------------------------------------------------------------------------------------------------------------- :   -
TN_TML1.3A   : ----------------------------------------------------------------------------------------------------------------------- :   -
DR_TML1.3B   : ----------------------------------------------------------------------------------------------------------------------- :   -
BT_12        : ----------------------------------------------------------------------------------------------------------------------- :   -
TN_MS4Ax1    : ----------------------------------------------------------------------------------------------------------------------- :   -
XT_MS4Ax1    : ----------------------------------------------------------------------------------------------------------------------- :   -
TN_MS4Ax4    : ----------------------------------------------------------------------------------------------------------------------- :   -
SP_out       : ----------------------------------------------------------------------------------------------------------------------- :   -
```

```
                   1080         *      1100         *      1120         *
MM_10       : ------------------------------------------------------------ :    -
HS_10       : ------------------------------------------------------------ :    -
BT_10       : ------------------------------------------------------------ :    -
OA_10       : ------------------------------------------------------------ :    -
HS_18       : ------------------------------------------------------------ :    -
BT_18       : ------------------------------------------------------------ :    -
MM_18       : ------------------------------------------------------------ :    -
MD_8B       : ------------------------------------------------------------ :    -
HS_8B       : ------------------------------------------------------------ :    -
BT_8B       : ------------------------------------------------------------ :    -
MM_8A       : ------------------------------------------------------------ :    -
OA_8A       : ------------------------------------------------------------ :    -
MM_13       : ------------------------------------------------------------ :    -
BT_13       : ------------------------------------------------------------ :    -
HS_13       : ------------------------------------------------------------ :    -
HS_12       : ------------------------------------------------------------ :    -
MM_12       : ------------------------------------------------------------ :    -
TG_MS4A15   : ------------------------------------------------------------ :    -
AC_MS4Ax1   : ------------------------------------------------------------ :    -
AC_MS4Ax2   : ------------------------------------------------------------ :    -
DR_17A.15   : ------------------------------------------------------------ :    -
DR_17A.3    : ------------------------------------------------------------ :    -
DR_17A.2    : ------------------------------------------------------------ :    -
DR_17A.5    : ------------------------------------------------------------ :    -
DR_17A.1    : ------------------------------------------------------------ :    -
DR_17A.12   : ------------------------------------------------------------ :    -
DR_17A.4    : ------------------------------------------------------------ :    -
DR_17A.10   : ------------------------------------------------------------ :    -
DR_17A.6    : ------------------------------------------------------------ :    -
DR_17A.11   : ------------------------------------------------------------ :    -
DR_17A.7    : ------------------------------------------------------------ :    -
DR_17A.8    : ------------------------------------------------------------ :    -
DR_17A.9    : ------------------------------------------------------------ :    -
DR_17A.13   : ------------------------------------------------------------ :    -
DR_17A.16   : ------------------------------------------------------------ :    -
DR_17A.17   : ------------------------------------------------------------ :    -
DR_17A.14   : ------------------------------------------------------------ :    -
DR_17B.1    : ------------------------------------------------------------ :    -
DR_17B.2    : ------------------------------------------------------------ :    -
DR_17C.2    : ------------------------------------------------------------ :    -
DR_17C.1    : ------------------------------------------------------------ :    -
OM_17C      : ------------------------------------------------------------ :    -
TN_MS4Ax3   : ------------------------------------------------------------ :    -
TN_MS4Ax2   : ------------------------------------------------------------ :    -
SA_MS4Ax1   : ------------------------------------------------------------ :    -
AT_MS4Ax1   : ------------------------------------------------------------ :    -
XT_MS4Ax2   : ------------------------------------------------------------ :    -
BT_1        : ------------------------------------------------------------ :    -
HS_1        : ------------------------------------------------------------ :    -
MM_1        : ------------------------------------------------------------ :    -
MD_1        : ------------------------------------------------------------ :    -
MD_15       : ------------------------------------------------------------ :    -
OA_15       : ------------------------------------------------------------ :    -
HS_15       : ------------------------------------------------------------ :    -
BT_15       : ------------------------------------------------------------ :    -
MM_15       : ------------------------------------------------------------ :    -
MM_5        : ------------------------------------------------------------ :    -
HS_5        : ------------------------------------------------------------ :    -
BT_5        : ------------------------------------------------------------ :    -
HS_14       : ------------------------------------------------------------ :    -
BT_14       : NCSSYQSSVQDTYHAYMSNKNSEQDVKQDTSTCSAVCKEGQSLASTSCGSKEREQSEDLG :  861
MM_2        : ------------------------------------------------------------ :    -
HS_2        : ------------------------------------------------------------ :    -
BT_2        : ------------------------------------------------------------ :    -
HS_6A       : ------------------------------------------------------------ :    -
HS_6E       : ------------------------------------------------------------ :    -
MM_6D       : ------------------------------------------------------------ :    -
MM_11       : ------------------------------------------------------------ :    -
MM_6B       : ------------------------------------------------------------ :    -
MM_6C       : ------------------------------------------------------------ :    -
HS_7        : ------------------------------------------------------------ :    -
MM_7        : ------------------------------------------------------------ :    -
BT_7        : ------------------------------------------------------------ :    -
HS_3        : ------------------------------------------------------------ :    -
MM_3        : ------------------------------------------------------------ :    -
MM_4B       : ------------------------------------------------------------ :    -
MM_4C       : ------------------------------------------------------------ :    -
MM_9        : ------------------------------------------------------------ :    -
MM_4D       : ------------------------------------------------------------ :    -
HS_4E       : ------------------------------------------------------------ :    -
HS_4A       : ------------------------------------------------------------ :    -
MM_4x       : ------------------------------------------------------------ :    -
AC_MS4Ax3   : ------------------------------------------------------------ :    -
BT_TM176B   : ------------------------------------------------------------ :    -
HS_TM176B   : ------------------------------------------------------------ :    -
MM_TM176B   : ------------------------------------------------------------ :    -
OA_TM176B   : ------------------------------------------------------------ :    -
TN_TML1.3B  : ------------------------------------------------------------ :    -
HS_TM176A   : ------------------------------------------------------------ :    -
BT_TM176A   : ------------------------------------------------------------ :    -
MM_TM176A   : ------------------------------------------------------------ :    -
DR_TMLx     : ------------------------------------------------------------ :    -
DR_TML1.2   : ------------------------------------------------------------ :    -
DR_TML1.3A  : ------------------------------------------------------------ :    -
TN_TML1.3A  : ------------------------------------------------------------ :    -
DR_TML1.3B  : ------------------------------------------------------------ :    -
BT_12       : ------------------------------------------------------------ :    -
TN_MS4Ax1   : ------------------------------------------------------------ :    -
XT_MS4Ax1   : ------------------------------------------------------------ :    -
TN_MS4Ax4   : ------------------------------------------------------------ :    -
SP_out      : ------------------------------------------------------------ :    -
```
