# Supplementary material for: Phylogenetic Analysis of the MS4A and TMEM176 Gene Families
Source: PLoS One. 2010 Feb 23;5(2):e9369. doi: 10.1371/journal.pone.0009369 (PMC2826416; doi:10.1371/journal.pone.0009369)
Supplement: Figure S3 — Alignment of subunits of long MS4A sequences. The long MS4A protein sequences were cut into their subunit components designated with the suffix A through F to denote their position relative to the amino terminus. Alignment of these subunits and human MS4A protein sequences was performed using ClustalX. (0.12 MB PDF) [file pone.0009369.s005.pdf]

```
                   *        20         *        40         *        60         *
HS_15   : MSAAPASNGVFVVIPPNNASGLCPPPAILPTSMCQPPGIMQFEEPPRGAQTPRATQPPDLRPVETFLTGEPKVL :  74
GG_15.A : -------------------------------------------------------------------------M :   1
GG_15.B : ------------RIITEVIPATDPRAAQLVSGSVQPTVSSFQISG--------------------FRRAQPKVL :  42

              80         *      100         *      120         *      140
HS_15   : GTVQILIGLIHLGFGSVLLMVRRGH-VGIFFIEGGVPFWGGACFIISGSLSVAAEKNH-TSCLVRSSLGTNILS : 146
GG_15.A : GTIQIMTGFMHIGFGIVL-TTLTNVYSSIFITG-EIPFLGGVSFIISGCLSIGAE-KSPTECAVKGSQATNIIS :  72
GG_15.B : GTIHIVTGIIHLCFGVILTCAENNSALSLPVAS-GVFFWLGVLLLVSGSLLVESE-KRENILLVKVCCVANAAV : 114

           *      160         *      180         *      200         *      220
HS_15   : VMAAFAGTAILLMDFGVTNRD----------------------------VDRGYLAVLTIFTVLEFFTAVIAMH : 192
GG_15.A : AIFALLGIVAFIIDLNFNGLYRS----------SDDYY-----SYLVLLAGNGISIVLLIFTILEFCIAVATAN : 131
GG_15.B : ILSSLVAMLIHTVAITHNIPGCSSSASMPLLVR-----QEWCFSAETKALSNGFDSILVLFGLLEFCTAVAALA : 183

                 *      240         *      260         *      280
HS_15   : FGCQAIHAQASAPVIFLP-----NAFSADFNIPSP-AAPAPPAYDNVAY-AQGVV--- : 240
GG_15.A : FWCRATRLSPNEAMLIVPSTTRVDLAVVQ------AELPQPPSYSEVMATTVTESGGV : 183
GG_15.B : FGCTAIKQYSYTRMVL------------------------------------------ : 199
```

```
                   *        20         *        40         *        60         *
HS_12    : --MMSSKPTSHAEVNETIPNPYPPGSFMAPGFQQPLGSINLENQAQGAQRAQPYGITSPGIFASSQPGQGNIQM :  72
MD_12.A  : -------------------------------------------------------------------------- :   -
MD_12.B  : EGDLDRAWPRLLLAAEGVSDREAEVLRLASASTGCRVSAGPGAGESTPKGGGPEGWDADGFPEPEPASPLPVGG :  74

              80         *       100         *       120         *       140
HS_12    : INPSVGTAVMN--------------FKEEAKALGVIQIMVGLMHIGFGIVLCLISFSFREVLG--FASTAVIGG : 130
MD_12.A  : -----------------------------MLQETAIQILIGLMHLGLGIILSLLSVHIDVLYSGYATLS-FVSG :  44
MD_12.B  : WALLVRRSSFPLGASTRDVLVLAPRSGVSSRTLSAIQISIGIFQLFLGILWF------------YLYLTQFADS : 136

            *       160         *       180         *       200         *       220
HS_12    : YPFWGGLSFIISGSLSVSASKELSRCLVKGSLGMNIVSSILAFIGVILLLVDMCINGVAG-QDYWAVLSGKGIS : 203
MD_12.A  : YPFWGGIGYICSGSFSVLAASSPTPYLVKSTLGMNIASAICALVGIILLLIDMILNGGIINQNYWAKVSGQGIS : 118
MD_12.B  : FLTYKTLIWNAKYP-----------------------------------------------LLSAFIASGVGIS : 163

                  *       240         *       260         *       280         *
HS_12    : ATLMIFSLLEFFVACATAHFANQANTTTNMSVLVIP----------NMYESNPVTPAS---SSAPPRCNNYSAN : 264
MD_12.A  : AMLVVFSLLELSINGKTSGFIMEMLMNKDLALGGGEL-------GAGKYRCPPTVLQQTQDPAQAQDPFGPDAN : 185
MD_12.B  : AVLFLFSVLELIISSISSHAGCQVVSSRTNQVAVAVAVPAHVAP-PMGIPQP--------AVNSLVYSNEVNSQ : 228

            300         *       320         *       340         *       360         *
HS_12    : APK----------------------------------------------------------------------- : 267
MD_12.A  : APAPARRHIKCAKGGHLQRKLAFCGNIFSAVLAVIGVILIIIEQCTEQEMSYKNFGRAAMGKLVSWLLLITSLG : 259
MD_12.B  : Q------------------------------------------------------------------------- : 229

                  380         *       400         *       420         *       440
HS_12    : -------------------------------------------------------------------------- :   -
MD_12.A  : EFSLTFTYIYHVMCNWDSLMSDLNDFYVEDKAEEEDEESLEEVMEEEVIKEWERKTSEELGRNMEKLEELEEVT : 333
MD_12.B  : -------------------------------------------------------------------------- :   -

                *       460
HS_12    : --------------------- :   -
MD_12.A  : EEEEEVEVVIKEKAKGRLRCC : 354
MD_12.B  : --------------------- :   -
```

```
                         *        20         *        40         *        60         *
HS_1   : ------------------------------------------MTTPRNSVNGTFPAEPMKGPIAMQSGPKP---- :  29
OA_1.B : WGDQQQQQQQRWNLKSAGSTSPRGLHQLDSGFPEETASSTIMMTPTGSVKGTGAVDSIKSTQVRQPSQRTVPRR :  75

             80         *       100         *       120         *       140         *
HS_1   : ---LFRRMSSLVGPTQSFFMRESKTLGAVQIMNGLFHIALGGLLMIPAGIYAPICVTVWYPLWGGIMYIISGSLL : 101
OA_1.B : ASR-------SVGPTQSFFMREAKPLGAVQIMNGLIHIALGGILMVPLGVYAPICITIWYPLWGGIMFIISGSLL : 143

                 160         *       180         *       200         *       220
HS_1   : AATEKNSRKCLVKGKMIMNSLSLFAAISGMILSIMDILNIKISHFLKMESLNFIRAHTPYINIYNCEPANPSEKN : 176
OA_1.B : VAAENSPSNILAKAKVGMNVISLFSAITGIIILIMDVFNITISHFFKMESLYLAKTSMPYINIYSCE------QS : 212

            *       240         *       260         *       280         *       300
HS_1   : SPSTQYCYSIQSLFLGILSVMLIFAFFQELVIAGIVENEWKRTCSRPKSNIVLLSAEEKKEQTIEI--KEEVVGL : 249
OA_1.B : GISQQYCLGMRAAFLGILSVMLTFTFLQNFVVAGITENEWRGLCSTARTVEKRHGSLIRHGPVPETRGPETLPTL : 287

                 *       320         *       340         *       360
HS_1   : TETSSQPKNEEDIEIIPIQEEEEEETETNFPEPPQDQESSPIENDSSP--------------- : 297
OA_1.B : TAQE--------EDTEISFPEPPQEKENRLGNLGSPCWSLQDGEGELGLCRKISGRRLRILRR : 342
```

```
                  *        20         *        40         *        60         *
HS_3   : ----------------------MASHEVDNAELGSASAHGTPGSETGPEELNTSVYHPINGSPDYQKAKLQVLGA :  53
MD_3.A : ----------------------MAQQIGESPQCVATSINNTHIMQLGKGVAARSSYNPLDESMKVLMGKQKVFGA :  53
MD_3.D : ----------------------MESQATTKETFSSFPQMGTISFPKGPTGTSVPFYKSKAYLQNFLKGEPKVLGS :  53
MD_3.C : ----------------------MESQATTKGTFSSFPQMGTISFPQGPTGTPVPFYKSKAYLQNFLKGEPKVLG- :  52
MD_3.F : ----------------------MESQATTKGTLSSFPQMGTISFPQGPIGTPVPLYKSKAYLQNFLKGEPKVLG- :  52
MD_3.E : ----------------------MESQATTKGTFSSFPQMGTISFPQGPIGTPVPFYKSKAYLQNFLKGEPKVLG- :  52
MD_3.B : ----------------------MESQATTKGTFSSFPQMGTISFPQGPIGTPVPLYKSKAYLQNFLKGEPKVLGT :  53
OA_1.A : ----------------------MESQPTPNGVFISFTQPGVHITQPGQAVASNSSNQPNGTLQKFLKGEPKVLGA :  53
OA_2.A : --------MYPTKRRFNITGNIMIPQTLPEGVLVIISSTGISISQTGQAGSAESPKKPPNKVKKILKEESRVLGV :  67
OA_2.B : MSTLRRSPALNTVLCTNIRHNIMRAQDVPRVPPVAFPRTGIDIFQSGQTVLPGSVPQPNSTLKKFLKGEPKILGA :  75

            80         *       100         *       120         *       140         *
HS_3   : IQILNAAMILALGVFLGSLQYPYHFQKHFFFFTFYTGY----PIWGAVFFCSSGTLSVVAGIKPTRTWIQNSFGM : 124
MD_3.A : LQILNGALILAIGIFLGSLQYVSQFPRNVFFMIFYTGY----PVWGAASFIISGSLSIVAEEKPTKNLVQSSFGM : 124
MD_3.D : VQIMIALMNFALGMIIILVQPRRYRHERFFLSTGYI-------FWGTAFFLVSGSLAIAAENKTTNTLVQSSLAM : 121
MD_3.C : --------------------------------------------------------------------VQSSLAM :  59
MD_3.F : --------------------------------------------------------------------VQSSLAM :  59
MD_3.E : --------------------------------------------------------------------VQSSLAM :  59
MD_3.B : VQIMIALMTFALGMIIILVQPRRYPHEHFFLSTGYI-------FWG-------------------TAFVQSSLAM : 102
OA_1.A : FQILIALVTLICGIVMLTSIYIPYVGFSGYSICG-------------------------------SIFIHGSLAM :  97
OA_2.A : LQILIGLILLFLGGILASVSVHPDLQPLLRYPFLG-----------AMLFIISGSLSVAAERKPTKLLGQGSLGM : 131
OA_2.B : IQILIALMILGTGVILICINQTQVFGSFLILYSGYPVWGTLLSKHSQAQAPMKEGSILRFRSGSVDSKVQNCLVM : 150

                160         *       180         *       200         *       220
HS_3   : NIASATIALVGTAFLSLNIAVNIQSLRSCHSS----------------SESPDLCNYMGSISNGMVSLLLILTLL : 183
MD_3.A : NIASATISLVGIIFLLINFVLINWEVRSCP------------------PESPNVCTLTSSVSIGLLSLMMILTVL : 181
MD_3.D : NTVSAVAAGLGIIFLSINLAMTDSSYYYCIH-----------------ESSYETCACGLAVLLGINVILLFLAIL : 179
MD_3.C : NTVSAVAAGLGIILLSINLAMTDSSYYFCTH-----------------ESSYETCACGLAVLLGINVILLILAIL : 117
MD_3.F : NTVSAVAAGLGIIILSINLAMNDSSYYSCIF-----------------ESPHETCVCGLAVLLGIGVILLILAIF : 117
MD_3.E : NTVSAVAAGLGIIFLSNKLSMADSSYYYCTH-----------------ESSYETCACGLAVLLGINVILLFLAIL : 117
MD_3.B : NTISTVAAGLGIIFLSINLAMNDSSYYYCYQ-----------------GTAQDTCVCGLAVLLGINVILLILAIL : 160
OA_1.A : NIISIVISGSGIIIISICLAINPYSLYHAPF------------------------------IYGLFSIELILTIL : 142
OA_2.A : NAISALAAATGFLILTINMAKIASNPDLCLHSFSHHPFASGHFDYDEPDQTEETVTATVKTEITEPERHLLVQHY : 206
OA_2.B : NIISSIAAVAGLIIISFYIAIDQQPRYVCHT-------------------SPKHMSTSCKVLSDISSVSGLSRKD : 206

             *       240         *       260         *       280         *       300
HS_3   : ELCVTISTIAMW--------------------CNANCCNSREE---------------------ISSPPNSV--- : 214
MD_3.A : ELCFTLSLTILG--------------------CKINCCESNEIG--------------------FSVEVRSILEG : 216
MD_3.D : EFAFSIAVSSFG--------------------CKATCCSQSGEALYP-----------PDS---SVVPIEQHHEG : 220
MD_3.C : EFAFSIAVSSFG--------------------CKATCCSQSGEALYP-----------PDS---SVVPIEQHREG : 158
MD_3.F : ELAFSLEVSSFG--------------------CKATCCSQSGVTIFM-----------PSP---SQVPVTEA--- : 155
MD_3.E : EFAFSIAGSSFGSPDPDLSLSSLVPALLTPAACSGSCFLLLASRLVPNLPALPACFCAPDPQIGSSVEERSILEG : 192
MD_3.B : EFAFSIAVSSFG--------------------CKATCCSQSG---IG-----------------SSVEVRSILEG : 195
OA_1.A : EFCITMSLSIYG--------------------CKAVCDDSNQASHSSASMAP-----IMASVSAPAASATAFLKT : 192
OA_2.A : DYFPHFILTFLD------------------EMCFLANSSLNRIKSMMLILT--------ILEFCIAAFLTCIIWK : 255
OA_2.B : QIHVSKSALEVE---------------------KDGDVVPRDIQRGG-----------------ARLGTKHCSKG : 243

                  *       320
HS_3   : -------------------- :   -
MD_3.A : GAPEIDPETLTTSGF----- : 231
MD_3.D : ----------TTSGF----- : 225
MD_3.C : ----------TTSGF----- : 163
MD_3.F : -------------------- :   -
MD_3.E : GAPEIDPETLTTSGF----- : 207
MD_3.B : GAPEIDPETLTTSGF----- : 210
OA_1.A : CEDGAGLQLLFSAR------ : 206
OA_2.A : ASDFRYNKIVTTSWFSSHVC : 275
OA_2.B : DSKES--------------- : 248
```
